# Soil Origin and Plant Genotype Modulate Switchgrass Aboveground Productivity and Root Microbiome Assembly

**DOI:** 10.1128/mbio.00079-22

**Published:** 2022-04-06

**Authors:** Pedro Beschoren da Costa, Gian Maria Niccolò Benucci, Ming-Yi Chou, Judson Van Wyk, Morgane Chretien, Gregory Bonito

**Affiliations:** a DOE Great Lakes Bioenergy Research Center, East Lansing, Michigan, USA; b Michigan State University, Department of Plant, Soil, and Microbial Sciences, East Lansing, Michigan, USA; Cornell University

**Keywords:** bioenergy crops, sustainability, 16S rRNA, ITS, random forest, rhizobiome, plant biomass

## Abstract

Switchgrass (*Panicum virgatum*) is a model perennial grass for bioenergy production that can be productive in agricultural lands that are not suitable for food production. There is growing interest in whether its associated microbiome may be adaptive in low- or no-input cultivation systems. However, the relative impact of plant genotype and soil factors on plant microbiome and biomass are a challenge to decouple. To address this, a common garden greenhouse experiment was carried out using six common switchgrass genotypes, which were each grown in four different marginal soils collected from long-term bioenergy research sites in Michigan and Wisconsin. We characterized the fungal and bacterial root communities with high-throughput amplicon sequencing of the ITS and 16S rDNA markers, and collected phenological plant traits during plant growth, as well as soil chemical traits. At harvest, we measured the total plant aerial dry biomass. Significant differences in richness and Shannon diversity across soils but not between plant genotypes were found. Generalized linear models showed an interaction between soil and genotype for fungal richness but not for bacterial richness. Community structure was also strongly shaped by soil origin and soil origin × plant genotype interactions. Overall, plant genotype effects were significant but low. Random Forest models indicate that important factors impacting switchgrass biomass included NO_3_^−^, Ca^2+^, PO_4_^3−^, and microbial biodiversity. We identified 54 fungal and 52 bacterial predictors of plant aerial biomass, which included several operational taxonomic units belonging to Glomeraceae and Rhizobiaceae, fungal and bacterial lineages that are involved in provisioning nutrients to plants.

## INTRODUCTION

Plants have evolved intimate associations with diverse microbial communities through processes of selection, drift, speciation, and dispersal (1). Host-microbe associations influence host plants’ yield and fitness, for example, by improving nutrient uptake, by mitigating disease and abiotic stress, and by regulating growth and development (2, 3). Fungi, bacteria, archaea, and other microorganisms that live on and within plant tissues can therefore be considered an extension of the plant phenotype, since their collective metabolism may aid the plant host in responding to environmental stressors (4–7). Consequently, characterizing plant-associated microbiomes and determining how they vary across plants and tissues is essential foundational research for leveraging host-microbe interactions for plant resilience, and to improve agricultural sustainability.

Plant microbiomes are known to vary between environments and soil types (8, 9), between plant species (10, 11), between different genotypes (12–14), and between compartments or individual plant tissues (2, 15, 16). Roots are specialized plant tissues that physically anchor plants to soils and also absorb water and nutrients from the soil, a function that is facilitated through their associated microbiome (7). Plant roots are known to secrete metabolites into soils, stimulating microbial activity and recruitment to the soil-root interface known as the rhizosphere (4, 17). Particular microbes are adapted to plants and develop closer association, colonizing the rhizoplane (root surface) as epiphytes, or by overcoming plant immunity to grow between and/or within the plant’s cells as endophytes (18). To fully exploit synergistic host-microbial interactions, it will be necessary to integrate breeding programs and cultivated genotypes with microbiome characterizations and applications.

It may not be surprising that the microbiome composition and structure of different plant genotypes varies. There is substantial genetic variation in plant immune response systems and other ecologically relevant phenotypic traits (13, 14). However, the magnitude of intraspecies host genetic control over its root microbiome has been reported to be relatively small and, for many plant species, is still largely unknown (9, 19). Further, significant genotype-environment interactions often limit the ability to estimate effects of genotype on plant microbiomes.

Switchgrass (*Panicum virgatum* L.) is a perennial grass native to the United States and is promising as a bioenergy crop due to its ability to produce high biomass, even in poor soils that are unsuitable for food crops (20). Other favorable traits of switchgrass as a biofuel crop are its wide geographic distribution, low susceptibility to pathogens, and high tissue quality for fuel conversion. Further, the cultivation of switchgrass can provide additional ecosystem services such as increasing soil biodiversity, water quality and carbon storage (21). Switchgrass genotypes fall into two major ecotypes: upland and lowland. Upland (e.g., Cave-in-Rock and Blackwell) ecotypes are adapted to mesic regions. They grow shorter and have higher tiller production than lowland ecotypes, and their nuclear condition is octoploid. In contrast, lowland ecotypes (e.g., Alamo and Kanlow) are adapted to hydric zones, grow taller with fewer tillers, are tetraploid, and are hypothesized to have descended from glacial refugia (20, 22). While scientific studies on switchgrass microbiome are increasing, the effect of switchgrass’s extensive genotypic and phenotypic variation on the rhizobiome (i.e., the microbial communities associated with the roots) are not well understood.

To address this knowledge gap, a common garden greenhouse experiment was carried out using six of the most common switchgrass cultivars, including both lowland and upland ecotypes, and switchgrass field soils from four geographical locations established by the Great Lake Bioenergy Research Center (GLBRC). Specifically, this research addresses the hypotheses that both plant genotype and soil origin will impact the rhizobiome community and plant biomass. We also hypothesize that particular soil characteristics and microbial taxa will be associated with higher plant biomass. In doing so, we assess how plant and soil factors interact to impact the assembly of root-associated fungi and bacteria in the switchgrass rhizobiome, and we determine how nonrandom plant-soil-microbe interactions impact switchgrass biomass. Microbial isolates from the switchgrass rhizobiome generated through this research will be available for future mechanistic validation on microbiome impacts on switchgrass biomass.

## RESULTS

### Sequencing results and overall community composition.

After bioinformatic analyses we obtained totals of 9,311,595 and 5,454,896 quality-filtered paired-end Illumina MiSeq ITS1 and 16S reads from 190 root and 40 soil DNA samples, respectively. These clustered into 2,782 fungal and 8,918 bacterial operational taxonomic units (OTUs) for the root samples and 4,798 fungal and 10,828 bacterial OTUs for the soil samples. Root samples had an average of 48,300.2 ± 18,083.9 SD (standard deviation) sequence reads for the fungi and 28,710.0 ± 12,595.9 SD sequence reads for the bacteria. Soil samples had an average depth of 84,375.5 ± 18,083.9 SD for the fungi and 162,847.5 ± 48,527.3 SD for the bacteria.

A total of 105 (1.29%) and 117 (0.56%) contaminant OTUs were removed from the ITS and 16S data sets, respectively (see Fig. S1 in the supplemental material). Sequences from the mock communities matched the number of taxa present in the mocks quite precisely. We recovered 14 OTUs of the 12 taxa present in the synthetic ITS mock community (with two OTUs for mock groups 1 and 10), and 7 OTUs instead of 8 present in the 16S mock community (Staphylococcus aureus the only missing taxa).

10.1128/mbio.00079-22.6 FIG S1 Contaminant OTUs and libraries depths. (A and B) Fungal and bacterial library depths for each sample; (C and D) contaminant OTUs removed from the dataset. Download FIG S1, PDF file, 0.3 MB. Copyright © 2022 Beschoren da Costa et al. 2022 Beschoren da Costa et al. https://creativecommons.org/licenses/by/4.0/ This content is distributed under the terms of the Creative Commons Attribution 4.0 International license.

### Alpha diversity.

**FIG 1:**
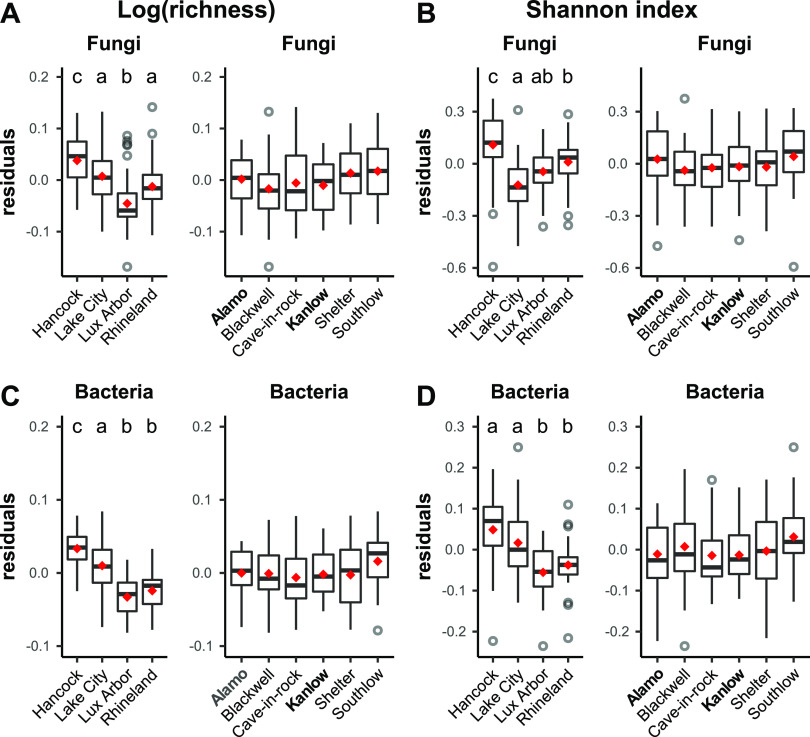
Alpha-diversity measures of microbial communities associated with switchgrass roots across soils and genotypes. Boxplots show the distribution of OTU richness (log transformed) and Shannon index soil across different plant genotypes for fungi (A and B) and bacteria (C and D). Lowland ecotypes are highlighted in boldface; upland ecotypes are unbolded. Values indicate the deviance residuals of log(richness) and the Shannon index generalized linear models after accounting for differential sequencing success: *model =* log(richness) ∼ readNo. Letters mark statistically significant differences evaluated with Tukey’s HSD after significant ANOVA (*P* ≤ 0.05). Boxplot outliers are reported as grey-colored open circles.

Fungal and bacterial OTU richness and Shannon index values of switchgrass roots differed significantly between soils but not between genotypes (Fig. 1; see also Fig. S2). In particular, regardless of plant genotype, a higher number of OTUs and more diverse microbiomes were found in switchgrass roots grown in Hancock soil compared to Lux Arbor and Rhineland (Tukey’s HSD, *P* ≤ 0.05), both for fungi (Fig. 1A and B) and bacteria (Fig. 1C and D). After accounting for differences in sequencing depth, general linear models (most parsimonious models included in this study) revealed that variations in OTU richness and Shannon index were statistically significantly explained by the experimental treatments (soil origin and plant genotype) (Fig. 1 and Table 1; see also Table S1 in the supplemental material). In addition to significant main effects, a significant genotype × soil interaction was found for fungal richness (Table 1; see also Table S1). In addition, soils from the pots with no-plants supported a higher diversity of fungi and bacteria compared to roots, and OTU richness was higher in Hancock and Lake City compared to Lux Arbor and Rhineland for bacterial communities but not in the fungal communities (see Fig. S3).

**TABLE 1:** Effect of genotype and soil origin on log(richness) and Shannon diversity index of fungal and bacterial OTUs, explained by glm models (generalized linear models)^*a*^

*Parameter*
	*Log(richness)*				*Shannon index*			
	*Sum Sq*	*Df*	*F value*	*P value*	*Sum Sq*	*Df*	*F value*	*P value*
Fungi								
Read no.	0.006	1	2.758	0.099	0.968	1	46.017	**<0.001**
Genotype	0.023	5	2.266	**0.050**	0.059	5	0.558	0.732
Soil	0.181	3	29.339	**<0.001**	1.363	3	21.597	**<0.001**
Soil: genotype	0.081	15	2.613	**0.002**				
Residuals	0.327	159				3.660	174	
Bacteria								
Read no.	0.067	1	97.346	**<0.001**	0.014	1	2.622	0.107
Genotype	0.004	5	1.254	0.286	0.037	5	1.430	0.216
Soil	0.152	3	72.994	**<0.001**	0.385	3	24.702	**<0.001**
Residuals	0.121	174			0.904	174		

a Only the most parsimonious models, selected through backward selection, are reported. “Read no.” refers to the number of total sequence reads obtained for each sample. Significant *P* values (*P* ≤ 0.05) are highlighted in boldface.

10.1128/mbio.00079-22.1 TABLE S1 Generalized linear models on alpha-diversity metrics, detailed results. Download Table S1, PDF file, 0.07 MB. Copyright © 2022 Beschoren da Costa et al. 2022 Beschoren da Costa et al. https://creativecommons.org/licenses/by/4.0/ This content is distributed under the terms of the Creative Commons Attribution 4.0 International license.

10.1128/mbio.00079-22.7 FIG S2 Generalized linear model diagnostic plots for fungal (A) and bacterial (B) OTU richness and for fungal (C) and bacterial (D) Shannon index. Download FIG S2, PDF file, 0.2 MB. Copyright © 2022 Beschoren da Costa et al. 2022 Beschoren da Costa et al. https://creativecommons.org/licenses/by/4.0/ This content is distributed under the terms of the Creative Commons Attribution 4.0 International license.

10.1128/mbio.00079-22.8 FIG S3 Differences between microbial communities across unproductive agricultural land experiment sites for soil and roots samples. Letters represent significant differences (*P* ≤ 0.05 after Benjamini and Hochberg correction) using pairwise Wilcoxon tests. Download FIG S3, PDF file, 0.03 MB. Copyright © 2022 Beschoren da Costa et al. 2022 Beschoren da Costa et al. https://creativecommons.org/licenses/by/4.0/ This content is distributed under the terms of the Creative Commons Attribution 4.0 International license.

### Beta diversity.

**FIG 2:**
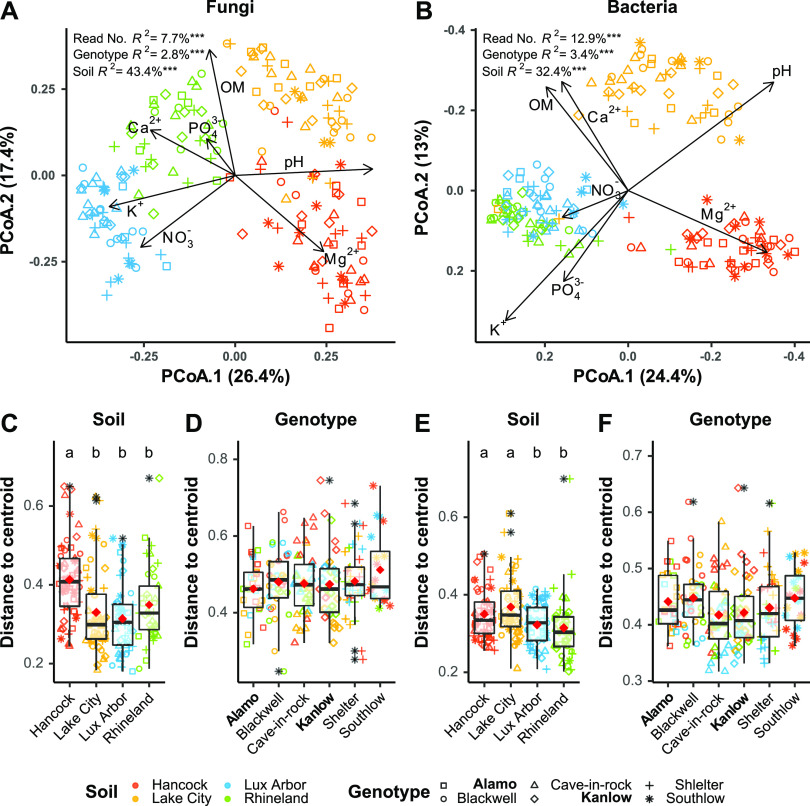
Community structure shown by principal coordinate analysis (PCoA) and distance to centroid distributions. (A) Fungal PCoA; (B) prokaryotic PCoA (axes were inverted to match samples organization in the ordination space of Fig. 2A); (C and D) dispersion around soil or genotype samples centroids for fungal communities; (E and F) dispersion around soil or genotype samples centroids for prokaryotic communities. The results from permutational multivariate analysis of variance are reported for significant factors. Lowland ecotypes are shown in boldface; upland ecotypes are unbolded. Letters mark statistically significant differences (*P* ≤ 0.05) evaluated with pairwise permutational ANOVA (perm. 9999) and *P* values corrected following Benjamini and Hochberg method. Boxplot outliers are reported as black-colored asterisks.

The switchgrass rhizobiome was structured primarily by soil origin and secondarily by plant genotype (Fig. 2A and B). Fitted vectors of the soil chemical parameters (i.e., pH, OM, Ca^2+^, Mg^2+^, K^+^, PO_4_^3−^, NO_3_^−^) correlated with specific soil origin in both fungal and bacterial microbiomes. For example, K^+^ and NO_3_^−^ were higher in Lux Arbor soil, while Mg^2+^ was higher in Hancock soils. Permutational multivariate analysis of variance (PERMANOVA; *P* ≤ 0.05) showed that about 8 and 13% of the variation for fungi (Fig. 2A) and prokaryotes (Fig. 2B) were significantly explained by differences in sequencing depth. However, soil origin was the main driver of community structure with about 44 and 32% of variation in community structures of fungi and bacteria, respectively, followed by genotype, which accounted for about 3% for both fungal and bacterial microbiome. Significant differences in dispersions (i.e., distance from sample centroids) were detected across soils but not genotype, with Hancock and Lake City showing higher dispersion compared to others (see Table S2A). This result demonstrates that underlying sample dispersion may contribute to significant differences in community structure between different plant genotypes within the same soil. When we separated samples from soils with average lower dispersions (i.e., Rhineland and Lux Arbor) from those with average higher dispersions (i.e., Hancock and Lake City), the effect of genotype (PERMANOVA, *P* ≤ 0.05) on root microbiome was significant only in the soils with higher dispersion but not in those with lower (see Table S2B).

10.1128/mbio.00079-22.2 TABLE S2 Permutational multivariate analysis of variance using distance matrices [adonis()], detailed results. Models were fitted using ungrouped and grouped soil samples. Soils were grouped according to high or low beta dispersion [see betadisper()]. Download Table S2, PDF file, 0.09 MB. Copyright © 2022 Beschoren da Costa et al. 2022 Beschoren da Costa et al. https://creativecommons.org/licenses/by/4.0/ This content is distributed under the terms of the Creative Commons Attribution 4.0 International license.

### Predictors and potential drivers of plant biomass.

**FIG 3:**
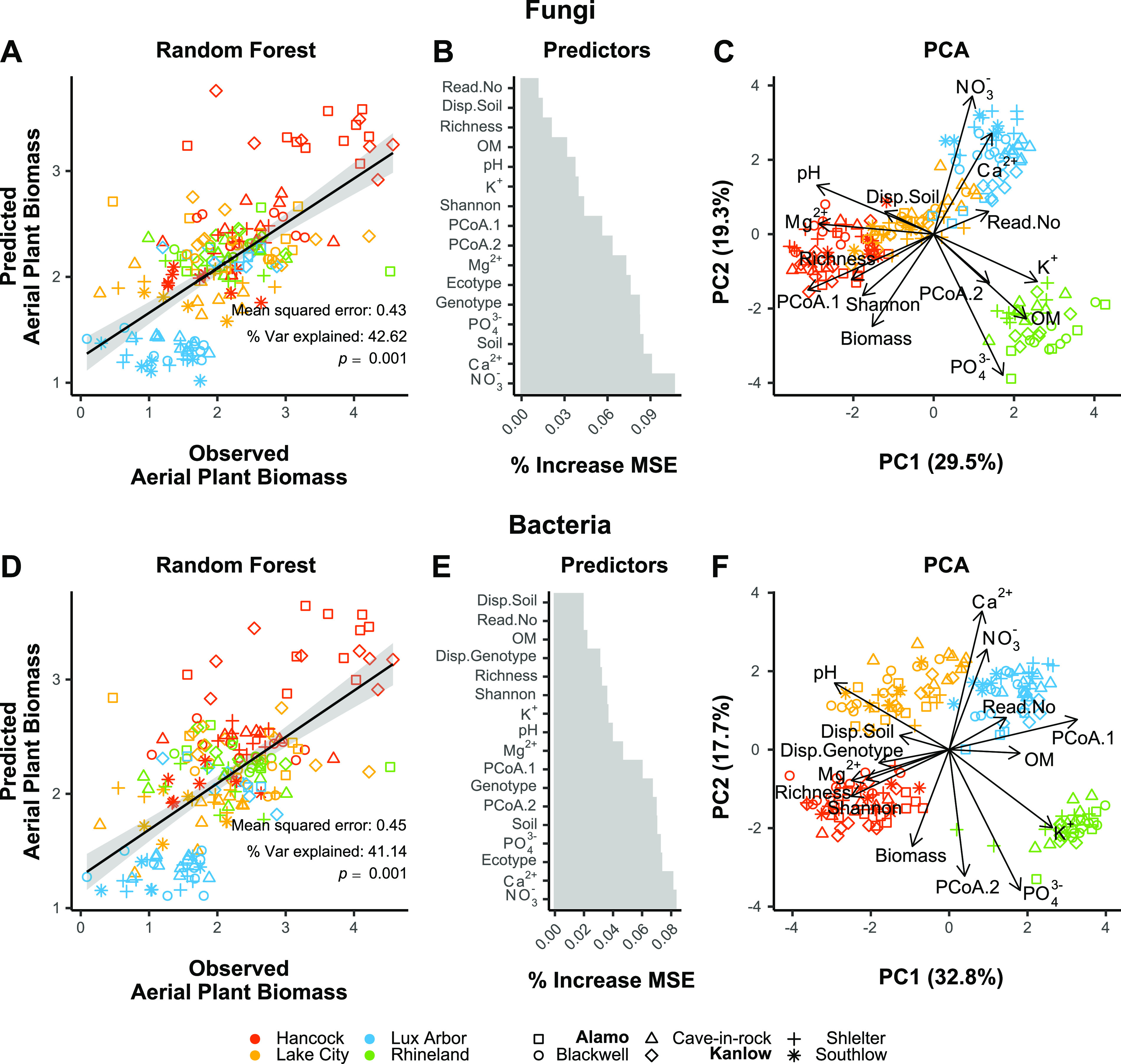
Random forest (RF) modes of aerial plant biomass using fungal and bacterial communities traits and soil chemical properties as predictors. A and D, RF regression lines; B and E, % increase MSE (mean squared error) of the selected predictors (important variables after 99 *Boruta* iterations) used for the RF models; C and F, PCA of predictors with loadings for fungal and bacteria communities, respectively. Predictors were all z-score transformed before running the models. Model errors, percent explained variance, and significance (perm. 9999) are also reported directly in the graphs. Lowland ecotypes are highlighted in boldface; upland ecotypes are unbolded. Abbreviations: Disp.Soil = multivariate dispersion for soil groups, Disp.Genotyep = multivariate dispersion for genotype groups, Read.No = number of total sequence reads obtained in each sample, PcoA.1 = first axis PCoA of Fig. 2, and PcoA.2 = second axis PCoA of Fig. 2.

Random Forest (RF) models were able to identify and rank the main environmental factors, microbiome diversity and structure properties, as well as specific OTUs that influence plant biomass for each of the soils (Fig. 3). Aboveground plant biomass was highest for plants grown in Hancock soil and lowest in Lux Arbor, with Rhineland and Lake City in the middle range. Models explained approximately 42 and 41% of the variation and were significant (*P* = 0.001 after 9,999 permutations), respectively, for fungi (Fig. 3A) and bacteria (Fig. 3D). Soil chemical properties, such NO_3_^−^, Ca^2+^, and PO_4_^3−^ were the most important model features for fungi (Fig. 3B) and bacteria (Fig. 3E), followed by community structure properties (e.g., PCoA.1 and PCoA.2 axes), treatments (ecotype, genotype, and soil), and finally alpha-diversity metrics (OTU richness and Shannon index). The importance of sequencing depth (the number of reads/library) in the models was very low for both fungi and bacteria. In addition, principle components analysis (PCA) ordinations (Fig. 3C and F) showed that some predictors were correlated. Therefore, the importance of individual predictors is difficult to assess. For example, higher plant biomass is correlated with higher fungal OTU richness, Shannon index, and community structure (PCoA.1), as well as to soil pH and Mg^2+^ content in Hancock soil. Similarly, Ca^2+^, NO_3_^−^, and K^+^ are correlated, i.e., higher in Lux Arbor soil samples, and their direction is opposite that of the aboveground plant biomass.

**FIG 4:**
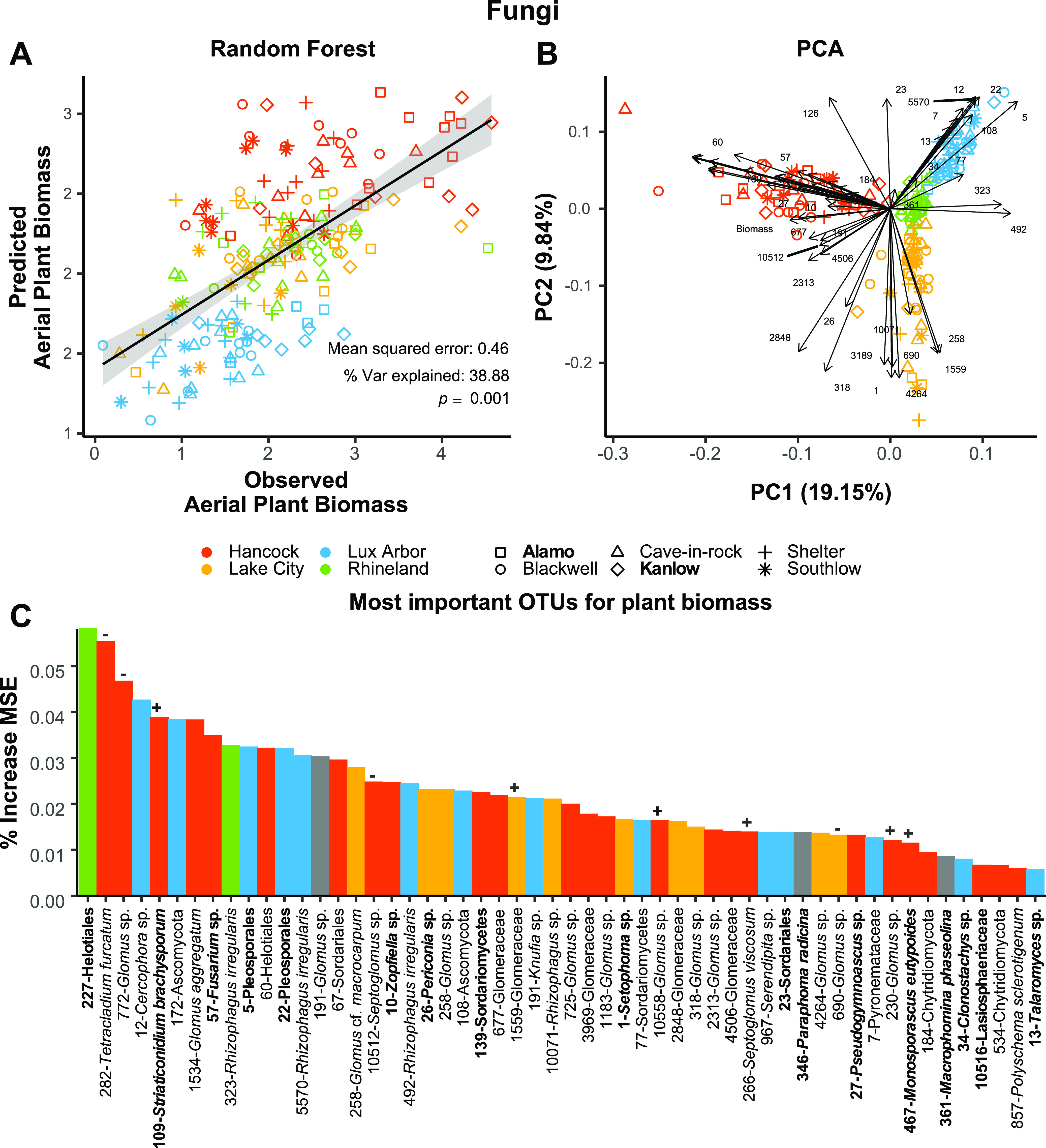
Random forest (RF) model of aerial plant biomass using 54 selected (important variables after selection with *Boruta*) fungal OTUs as predictors. (A) RF regression lines; (B) PCA of predictors with loadings, (C) percent increase MSE (mean squared error) of the selected predictors used for the RF models. Model errors, percent explained variance, and significance (perm. 9999) are also reported directly in the graphs. Bar colors in panel C represent the association of an OTU with a specific soil (*P* ≤ 0.05 after Benjamini and Hochberg correction), “+” and “–” represent OTUs that are positively or negatively correlated with aerial plant biomass in linear models (see Table S3B), and boldface OTU names represent a match to our isolate collection. Not all OTU numbers are present to avoid overlapping, but they can be seen in a larger plot in Fig. S4A. Lowland ecotypes are shown in boldface; upland ecotypes are unbolded.

**FIG 5:**
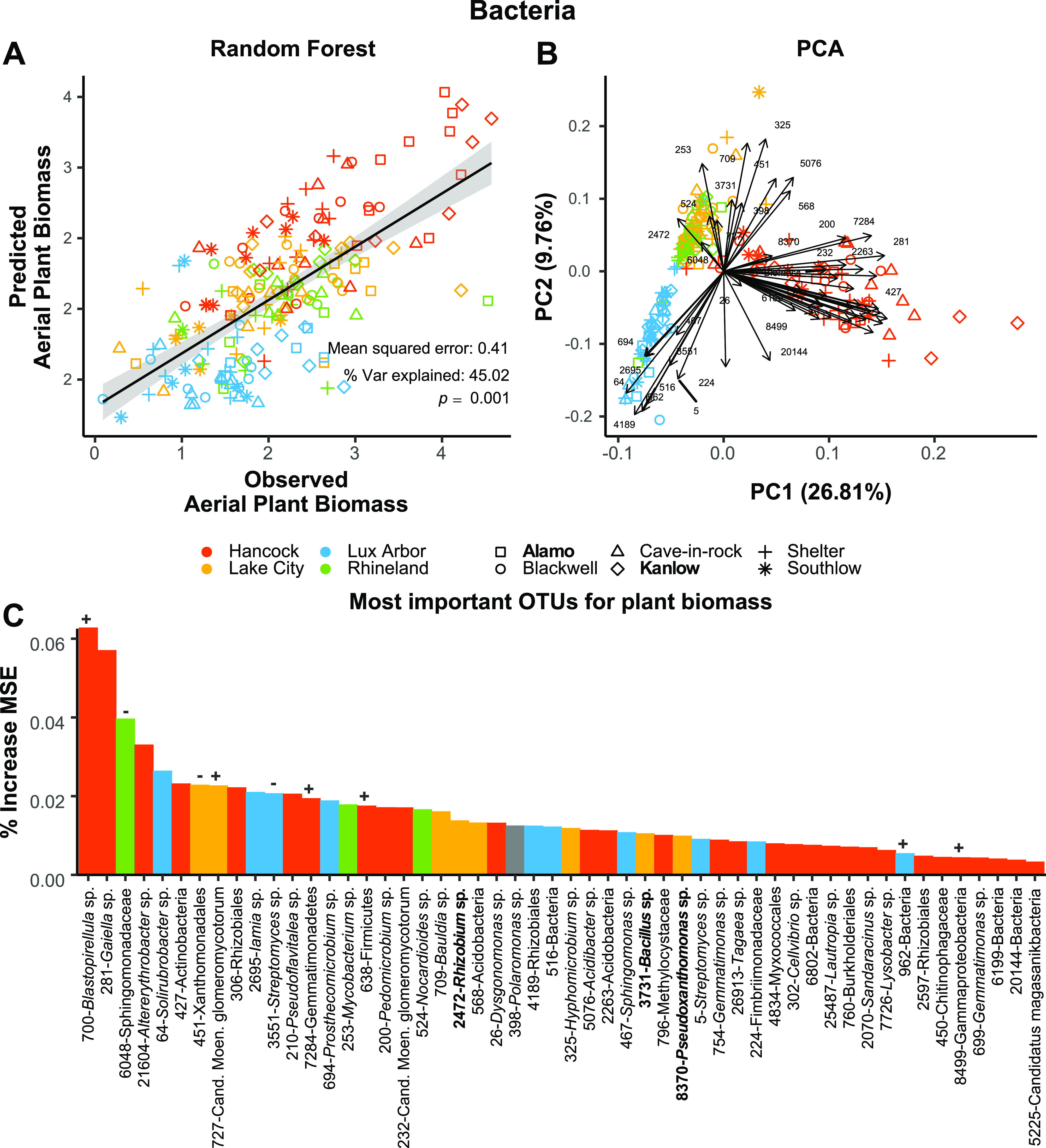
Random forest (RF) model of aerial plant biomass using 52 selected (important variables after selection with *Boruta*) bacterial OTUs as predictors. (A) RF regression lines; (B) PCA of predictors with loadings, (C) percent increase MSE (mean squared error) of the selected predictors used for the RF models. Model errors, % explained variance, and significance (perm. 9999) also reported directly in the graphs. Bar colors in panel C represent the association of an OTU with a specific soil (*P* ≤ 0.05 after Benjamini and Hochberg correction), “+” and “–” represent OTUs that are positively or negatively correlated with aerial plant biomass in linear models (see Table S3B), and boldface OTU names represent a match to our isolate collection. Not all OTU numbers are present in panel B to avoid overlapping, but they can be seen in a larger plot in Fig. S4B. Lowland ecotypes are shown in boldface; upland ecotypes are unbolded.

Random Forest models allowed us to select 54 (Fig. 4A; see also Fig. S4A) and 52 (Fig. 5A; see also Fig. S4B) top OTU predictors of plant biomass among 2,782 fungal and 8,918 bacterial OTUs to explain 39 and 45% of the variation in plant biomass. Arbuscular mycorrhizal fungi (AMF) were prevalent among these. Principal component analysis showed a correlation among OTUs belonging to the same lineage with a certain degree of specificity for certain soils (Fig. 4B and Fig. 5B). For example, *Glomus* and *Septoglomus* OTUs accounted for about 30% of all the 54 OTUs selected by RF, but the AMF taxa *Septoglomus viscosum* (OTU 266), *Glomus* sp. (OTU_230), and a *Glomeraceae* OTU (OTU_725) had longer vectors (i.e., greater importance) in the PCA plot (Fig. 4B) and were correlated with Hancock soils. In contrast, OTUs in the *Rhizophagus* genus pointed toward Lux Arbor and Rhineland and accounted for ∼7% of the 54 RF predictors. Other predictors of plant biomass were *Serendipita* and some less-well-characterized endophytes, including *Cercophora*, *Periconia*, and others associated with plant pathogenicity such as Fusarium, *Setophoma*, and *Zoopfiella* (Fig. 4C).

10.1128/mbio.00079-22.3 TABLE S3 (A) Fungal and bacterial random forest predictors (OTUs) of plant biomass. (B) Linear relationship between selected OTUs and aerial plant biomass. Download Table S3, PDF file, 0.5 MB. Copyright © 2022 Beschoren da Costa et al. 2022 Beschoren da Costa et al. https://creativecommons.org/licenses/by/4.0/ This content is distributed under the terms of the Creative Commons Attribution 4.0 International license.

10.1128/mbio.00079-22.9 FIG S4 Larger view of the PCA ordination of the top important fungal (A) and bacterial (B) OTU predictors of aerial plant biomass presented in FIG 4B and FIG 5B of the main text and Spearman correlation between aerial plant biomass, community metrics and chemical variables (C). Data were z-score transformed before correlation. Download FIG S4, PDF file, 0.05 MB. Copyright © 2022 Beschoren da Costa et al. 2022 Beschoren da Costa et al. https://creativecommons.org/licenses/by/4.0/ This content is distributed under the terms of the Creative Commons Attribution 4.0 International license.

Nearly 48% of the bacterial OTUs predictive of plant biomass belonged to the class *Proteobacteria*. *Alphaproteobacteria* (29%) and *Gammaproteobacteria* (18%) generally had longer vectors in the bacterial PCA plot (Fig. 5B) compared to other taxa. *Alphaproteobacteria* were present in nearly every soil, while *Gammaproteobacteria* were more concentrated in Hancock soil. Interestingly, two predictors of plant biomass belonged to the uncultured “*Candidatus* Moeniplasma Glomeromicotorum,” a mycoplasma-related endobacterium associated with arbuscular mycorrhizal fungi. *Rhizobiales* OTUs were the most common among the bacterial predictors of plant biomass (see Table S3A).

Among all the microbial predictors of plant biomass identified by random forest models, neutral models indicate that most were nonrandomly recruited (see Table S3A). It is worth nothing that about 73.3% of all *Glomeraceae* OTUs, which were predominant among the fungal RF predictors, were above the model prediction (i.e., they were selected by the host), 17.4% were as predicted (i.e., neutral), and 4.4% were below (i.e., dispersally limited) the prediction in the fitted neutral model (see Table S3A). In addition, a number of these identified predictors of plant biomass matched our collection of culture isolates (visualized as darker bars on Fig. 4 and 5C; see also Table S3A). Significant positive and negative associations between the important microbial predictors and switchgrass biomass were assessed through linear regression (see Table S3B). For the 54 fungal predictors, six significantly positive-regressed and five negative-regressed with biomass (see “+” or “–” in Fig. 4C). Among the 52 bacterial predictors, six significantly positive regressed and four negative regressed with biomass (see “+” or “–” in Fig. 5C). The selected predictive OTUs identified by RF models were also examined to determine whether they were skewed toward the roots of any particular soil. The results indicated that predictive fungal and bacterial OTUs were indicator taxa across different soils and not skewed toward any specific soil.

### Correlating the rhizobiome with plant phenotypic and soil chemical properties.

Correlations between microbial communities, switchgrass phenotypic traits and soil chemistry were all significantly (*P* ≤ 0.001) indicative of a three-way plant-soil-microbe interaction (Table 2; see also Table S4). Mantel tests results were consistent with diversity analysis results showing that soil (and the inherent soil properties) explained most of the microbial assemblage diversity, and plant genotype came next. Plant phenotype had a minor but significant correlation with soil chemistry, with an *R* value lower than that of microbe-plant phenotypic correlations.

**TABLE 2:** Three-way correlations of bacterial and fungal communities, plant phenotypic traits, and soil chemical properties according to the Mantel test

Correlation	Mantel statistic	
	*R*	*P* value
Bacteria vs plant phenotype	0.171	<0.001
Bacteria vs soil chemistry	0.3078	<0.001
Fungi vs plant phenotype	0.1438	<0.001
Fungi vs soil chemistry	0.2904	<0.001
Plant phenotype vs soil chemistry	0.0755	<0.001

10.1128/mbio.00079-22.4 TABLE S4 Mantel tests correlation between fungal communities and plant phenotype traits, detailed results. Download Table S4, PDF file, 0.05 MB. Copyright © 2022 Beschoren da Costa et al. 2022 Beschoren da Costa et al. https://creativecommons.org/licenses/by/4.0/ This content is distributed under the terms of the Creative Commons Attribution 4.0 International license.

### Diversity of isolated microorganisms.

We accessioned 361 fungal isolates that were classified to 48 different genera and matched 66 fungal OTUs at 97% similarity and 372 bacterial isolates that were classified to 32 different genera and matched 72 bacterial OTUs at 97% similarity. These were isolated from 112 individual plants, including at least three samples for each genotype in each soil. Plants with the greatest height were targeted for microbial isolation, including all of the 57 plants from Hancock. Fusarium accounted for 53% of the isolated fungi, followed by *Setophoma* with 8.6%, while Pseudomonas accounted for 25% of isolated bacteria, followed by *Agrobacterium* with 15.4% (see Fig. S5A and B).

10.1128/mbio.00079-22.10 FIG S5 Genus rank balloon plot showing occurrence of fungal (A) and bacterial (B) cultures isolated from soil and genotype samples. The size of the circles indicates the number of isolates from that genus sourced from a single pot. The color of the circles indicates the dry weight of the plant the isolate was sourced from, normalized to the average plant dry weight of each genotype in each soil. Genus rank accumulation curves of fungal (C) and bacterial (D) cultures isolated from soil and genotype samples. Media with antioxidant amendments are indicated with “+Antioxidant.” The vertical black line marks 50 isolates for fungi and 31 isolates for bacteria. Lowland ecotypes are shown in boldface. Download FIG S5, PDF file, 0.1 MB. Copyright © 2022 Beschoren da Costa et al. 2022 Beschoren da Costa et al. https://creativecommons.org/licenses/by/4.0/ This content is distributed under the terms of the Creative Commons Attribution 4.0 International license.

We tested whether amending an antioxidant mix to cultures could increase diversity of isolated bacteria and fungi. We found that it could, depending on the isolation media that was used. As can be seen in the accumulation curves (see Fig. S5C and D), the same isolation effort can provide a higher number of different genera if antioxidants are used, accounting for 1.7 extra genera for 50 isolates in 1% malt extract agar (MEA) and 2.4 extra genera for 50 isolates in 0.1% MEA. In addition, antioxidant amendments in MEA reduced the prevalence of Fusarium from 46 to 30%. Dilute MEA (0.1%) provided similar results, with 2.4 extra genera for 50 isolates and reducing Fusarium prevalence from 57 to 50%. Antioxidant amendments also increased the diversity of culturable bacteria in LGI-P (Liquid Glucose Ivo Pernanbuco) media, with 4.5 extra genera for 31 isolates and reducing Pseudomonas prevalence from 35 to 18%. For LGI media, the increase induced by antioxidants was of only 0.75 genera per 31 isolates, while the Pseudomonas prevalence increased from 13 to 20%. For NFB media, however, the antioxidant amendments reduced the number of culturable genera by 0.8 per 31 isolates and increased the prevalence of Pseudomonas from 29 to 32%. Cultivation of *Rhizobium* was also affected by the addition of antioxidants. An approximate 3.7% of strains in regular media were identified as *Rhizobium*, while 5.5% of strains in antioxidant amended media identified as *Rhizobium*, a relative increase of almost 50%. Ultimately, 58 different genera were defined for 425 isolates from regular growing conditions, and 50 different genera were defined for 240 isolates from antioxidant amended conditions. The representative sequences of the isolated microbes are available in the supplemental material.

## DISCUSSION

### Plant genotype has a negligible effect on switchgrass rhizobiome.

Plant genotype impacts on the plant microbiome have been challenging to study because of confounding interactions between genotype and the environment. In this study, we used a common garden design to evaluate the effect of switchgrass genotype and soil origin on fungal and bacterial rhizobiome members and identify microbiome taxa correlated with greater plant biomass. Our results demonstrate that plant genotype has a significant effect on the root microbiome structure but that the effect was small and driven by samples in soils having higher beta diversity and heterogeneity across samples. However, we found a significant soil × plant genotype interaction for fungal richness, suggesting that richness differences between genotypes change as soil factors change and that the host genotype-microbe interaction is soil dependent.

Previous research has found weak effects of host genotype (23, 24). For example, a significant interaction effect of soil type and genotypes was found for the soybean rhizosphere microbiome diversity, with soil type predominantly shaping the community assembly and host genotype only finely tuning the recruitment process (24). In Medicago truncatula, bacterial diversity was driven by significant soil origin effects in the rhizosphere and significant plant genotype effects in the root endosphere, with strong compartment × soil origin and compartment × plant genotype interactions reported (13).

### Soil origin is a major driver of fungal and bacterial communities in switchgrass roots.

We found that soil origin was the main driver of community structure in both bacteria and fungi, as samples grouped primarily by soil origin in the ordination space. In contrast, genotype significantly explained only a small position of the variance in the data. However, we understand soil origin encompasses many factors and constraints, including microbial biodiversity, soil physical and chemical factors, as well as the interactions of all of these factors. In addition, we homogenized field soil with sand in order to establish well-draining biological replicates for our greenhouse study; thus, the structure of the soil was disrupted. The microbial succession in the recovery phase of this disruption is a variable that we did not explicitly account for in this study.

Interestingly, plant genotype effects were significant in soils that showed high average heterogeneity (i.e., multivariate dispersion) but not in soils that showed low sample heterogeneity. These results indicate that certain plant genotypes are able to recruit higher microbial diversity in some soils but not others. This response is likely due to inherent soil physiochemical constraints on the microbial activity. Microbe-microbe interactions and soil factors, including soil type, pH, and organic matter, are known to influence microbial recruitment in the rhizosphere (4, 25). Bacteria may be better able to disperse and contact roots when water is freely available, while fungi are filamentous and are able to traverse from bulk soil to the root surface. Thus, fungi may be less dependent on small variations of soil moisture for dispersal compared to bacteria.

### Plant biomass is predicted by soil macronutrients, plant ecotype, and microbial diversity.

Random forest models were used to predict plant biomass from soil, plant and microbiome data. Major predictors of plant biomass identified through this approach were soil macronutrients. For example, NO_3_^−^ showed a slight negative and PO_4_^3−^ and Mg^2+^ showed a positive correlation to aboveground plant biomass (see Fig. S4C). Other variables that predicted plant biomass were soil origin, switchgrass ecotype and genotype, as well as microbial community diversity and structure. In fact, the roles of soil chemical properties, as well as microbial diversity and structure, are inseparable factors that are well known to significantly influence plant growth and development. Previous studies have suggested the importance of soil chemical properties in shaping microbial diversity and community structure, especially nitrogen, phosphorus, and pH (26–28), and the resulting belowground microbiome then iteratively affects the plant productivity (29–31). This soil-microbe-plant three-way interaction was again demonstrated in our study. What is special to our case is that the microbial beneficial effects seemed to overcome the soil nutritional effect reflected in plant biomass production, where higher NO_3_^−^ and K^+^ in Lux Arbor soil was coupled with both lower microbial diversity and plant biomass. It is likely that the activity of microbe-mediated nutrient acquisition in switchgrass grown in Lux Arbor soil was lower than switchgrass grown in other soils and resulted in the differential plant nutrient availability and biomass production. Mechanisms for how adaptive microbial communities can help plants acquire nutrients such as nitrogen and phosphorus has been extensively studied, with nutrient solubilization or mineralization via efflux of anions or protons, releasing enzymes and engaging ligand-exchange reactions identified as being important (32, 33). The relatively even indicator taxa selection across all soil types suggests that microbial taxa predictive of biomass predictions were likely not due to coincidental correlation between biomass and the selective microbiome resulting from any one specific soil.

### Switchgrass microbiome and isolated fungal and bacterial diversity.

This study underscores the important effect of arbuscular mycorrhizal fungi on switchgrass plant biomass. Nearly half of the OTUs selected by random forest among all OTUs predictive of aerial plant biomass were arbuscular mycorrhizal fungi (AMF) in *Glomeraceae*. It is well known that AMF promotes plant growth by facilitating nutrient acquisition, particularly phosphorus (34, 35). In addition to the AMF, one OTU belonging to the plant-growth-promoting *Serendipita* (*Sebacinales*) was also selected by the random forest models. *Serendipita* are a known switchgrass growth promoter taxon that enhance nutrient availability and stress tolerance of host plants (36–38). In our study, *Glomeraceae* OTUs were more abundant and diverse than *Sebacinales* OTUs, supporting previous findings showing that *Sebacinales* to be less diverse, common, and abundant than Glomeromycota in the switchgrass system (39). Interestingly, a Mollicute-related endosymbiont (MRE) of AMF (“*Ca.* Moeniiplasma glomeromycotorum”) was also identified as being important to switchgrass biomass. Perhaps, MRE are a biomarker for AMF or, alternatively, they may be adaptive elements in plant microbiomes. Unfortunately, AMF and their MRE symbionts are both obligate biotrophs. Therefore, tests on their impact on plant biomass are more difficult to conduct directly with these taxa. However, among the effective biomass fungal predictors identified by random forest models, 16 of them are represented in the collection of switchgrass isolates obtained in this research. Of these, *Striaticonidium brachysporum* and *Monosporascus eutypoides* may yield special interests since they were further selected in the linear regression model showing significant and positive association with switchgrass biomass. Interestingly, many closely related species or species in genera *Striaticonidium* and *Monosporascus* are known to be plant pathogens; however, many are plant endophytes, and their functional relationship with plants may differ between species or hosts (40–42). Further investigations with switchgrass isolates will allow for direct tests on beneficial effects and mechanisms of switchgrass biomass accumulation under different soil conditions, such as varied levels of microbial diversity or plant nutrients.

The bacterial isolation effort utilizing antioxidants and N-free media resulted in a large number of strains belonging to the *Rhizobium* genera, a group of bacteria that were also predictive of plant biomass. N-free semisolid media has been shown to be very successful at retrieving rhizobia from nonleguminous plants (43), while the addition of antioxidants should alleviate oxidative stress, possibly rescuing bacteria from the viable but nonculturable condition (44, 45). Given that the unproductive agricultural lands that compose the soils in this study are by definition nutrient poor (20), one could expect that associative N-fixing bacteria could be playing a relevant role in nitrogen nutrition for switchgrass plants. Interestingly, NO_3_^−^ was predictive of lower switchgrass biomass in the RF model, indicating, switchgrass may prefer NH_4_^+^ or is not N limited. Switchgrass had not been selected to respond to fertilizer application and instead may rely on associative N fixation or other episodic microbial associations for maintaining nutrition (46, 47). Finally, there were four *Rhizobiales* OTUs highlighted as relevant in the random forest model, one being a *Rhizobium* that matches a strain in our collection, *Rhizobium* sp. strain GLBRC894. Further studies with fungal and bacterial isolates from switchgrass will allow for more direct tests on pairwise or consortium effects microbiome members on plant traits of interest, including growth, nutrition, and stress resilience.

### Conclusion.

In conclusion, this research showed significant soil effects and minor genotypic effects on switchgrass root-associated microbial recruitment among six switchgrass cultivars belonging to two ecotypes and grown across four soils. Both soil chemical properties and root-associated microbiome were significant factors in explaining aerial biomass variation. While soil fertility is known to be important to plant growth, microbial factors had a stronger effect on plant biomass, reinforcing the significant role of microbes in shaping plant growth and development. Plant-beneficial microbes, including AMF and *Rhizobiales*, were identified as potential switchgrass biomass promoters in this study. Further directed studies made possible with microbial isolates generated through this work, including *Striaticonidium brachysporum* and *Monosporascus eutypoides*, and putative N-fixing bacteria in the *Rhizobiales* can be used to validate results and facilitate bioenergy crop productivity in unproductive agricultural land for a more sustainable bioenergy output.

## MATERIALS AND METHODS

### Preparation of microcosms.

Samples of the top 10 to 15 cm of field soil surrounding the switchgrass plots of marginal land experiment sites from GLBRC were collected at Hancock (Hancock, WI), Lake City (Lake City, MI), Lux Arbor (Delton, MI), and Rhinelander (Rhinelander, WI) and stored at 4°C for ∼4 weeks prior to planting. Soil characteristics are available in Kasmerchak and Schaetzl (48) and are summarized in Table S5 in the supplemental material.

10.1128/mbio.00079-22.5 TABLE S5 General characteristics, taxonomy, and location of the soils used to build the microcosms. Download Table S5, PDF file, 0.03 MB. Copyright © 2022 Beschoren da Costa et al. 2022 Beschoren da Costa et al. https://creativecommons.org/licenses/by/4.0/ This content is distributed under the terms of the Creative Commons Attribution 4.0 International license.

Approximately 38 L of soil from each of these sites was sieved to remove organic and mineral debris, mixed 1:1 with autoclaved coarse sand, homogenized, and filled into 1-L pots. Sand was sterilized by first hydrating 5 L of sand with 1 L of ddH_2_O and then autoclaving this twice at 121°C for 2 h, with a 24-h cooling interval between autoclave cycles.

Switchgrass seed of the lowland ecotypes Alamo and Kanlow and the upland ecotypes Cave-in-rock, Shelter, Blackwell, and Southlow were obtained from Native Connections (Kalamazoo, MI). To remove the seed microbiome, ∼2-mL portions of seeds were placed in a sterile 15-mL tube, and dry seeds were exposed to a 55°C water bath for 1 h to prevent the viability of fungal seed endophytes (49). The seeds were then surface sterilized in a four-step process that included washing with 95% ethanol for 1 min, washing with 3% active household bleach for 5 min, washing with 95% ethanol again for 1 min, and finally rinsing three times with sterile ddH_2_O. Approximately 40 seeds were then spread in plates with sterilized kimwipes saturated with sterile water and left for 2 weeks under artificial lights to pregermination. Five pregerminated seeds of similar size from all six genotypes were planted in pots containing four soils and were then thinned to one plant per pot 2 weeks after planting. There were 28 treatments, including no-plant controls for all soils, and 10 replicates per treatment. Plants were grown for 4 months in a temperature-controlled greenhouse at Michigan State University. Plant traits, including height, the number of leaves, the number of tillers, and the flowering stage, were measured at 12 and 16 weeks after planting. Other traits, including the root length, the number of nodes, and the plant aerial part dry weight, were measured 16 weeks after planting. Soil pH, PO_4_^3−^, K^+^, Ca^2+^, Mg^2+^, organic matter (OM), and NO_3_−N contents for each treatment were measured by the Michigan State University (MSU) Soil Test Laboratory.

Roots were harvested from each plant for microbial isolation and DNA extraction. Roots were rinsed with a 0.5% Tween 20 wash to remove soil debris and then rinsed three times with sterile ddH_2_O. Washed roots were then subsampled for isolation and DNA extractions. Root tissue for DNA extraction was flash-frozen with liquid nitrogen and stored in −80°C until lyophilization and bead beating. Soil from plant-free pots was homogenized, flash-frozen with liquid nitrogen, and stored in −80°C until lyophilization and DNA extraction.

### Isolation of microorganisms and antioxidant treatments.

To isolate fungi from switchgrass roots, soil-free roots were first washed as described above. They were then surface sterilized by shaking the roots for 1 min in ethanol 70%, then for 2 min in 50% household bleach (or final 3.75% sodium hypochlorite), and then for an additional 1 min in ethanol 70% and finally rinsed five times with sterilized ddH_2_O (50). Roots were then cut in 2- to 5-mm pieces with sterilized scissors. For fungus cultivation, root fragments were gently submerged into solid 1% malt extract agar (MEA; 10 g/L malt extract, 10 g/L agar, 2.5 g/L yeast extract) and 0.1% MEA, which were both amended with the B vitamins thiamin (1 mg/L) and biotin (0.5 mg/L) and the broad-spectrum antibiotics rifampicin (50 mg/L) and chloramphenicol (100 mg/L). An antioxidant mix was applied to part of the isolation cultures to assess whether it would increase microbial culturability by reducing oxidative stress. This antioxidant mix was composed of a filter-sterilized mixture of catalase (3.5 mg/mL), peroxidase (0.7 mg/mL), sodium ascorbate (50 mg/mL), and sodium pyruvate (50 mg/mL) and was kept in ice during preparation and application. For fungal cultures, a 10-μL droplet of the antioxidant mix was applied directly on root fragments immediately after incubation. Cultures from roots were incubated at room temperature for 3 to 6 days before a small portion of media with mycelia was transferred to fresh solid media to establish pure isolates. Cultures were archived long-term in vials by transferring four to six agar slabs to 10 mL of sterile ddH_2_O.

Bacteria were isolated from switchgrass roots on the nitrogen-free media New Fábio Pedrosa (NFB), Liquid Glucose Ivo (LGI), and Liquid Glucose Ivo Pernanbuco (LGI-P) to select for putative diazotrophs, as described previously (50). Briefly, surface-sterilized switchgrass roots were resuspended in 25 mL of sterile NaCl 0.85% and left to shake overnight at 4°C. For bacterial cultures, 100 µL of the antioxidant mix (described above) was applied in 10 mL of semisolid NFB, LGI, and LGI-P media in 20-mL vials. Then, 100 µL of the antioxidant mix was spread in solid N-free plates immediately before streaking.

### Identification of cultured strains.

Fungal DNA was extracted by incubating a small portion of mycelia in 20 µL of an extraction solution (filter-sterilized 1 M Tris [1 mL/L], 0.186 g/L KCl, and 37 mg/L EDTA and then adjusted to pH 9.5 to 10.0 with 1 M NaOH) for 10 min before lysing at 95°C for 10 min. Directly after extracting the DNA, 60 μL of filter-sterilized bovine serum albumin 3% was added to each tube. PCR amplification was carried out with the primer set ITS1F/LR3 at 10 µM, 0.375 µL each per reaction; Platinum Green Hot Start PCR Master Mix (2×; Invitrogen), 6.25 µL per reaction; 3 µL of ultrapure water per reaction; and 2 µL of DNA extract per reaction. The reaction was conducted in a thermocycler beginning with 3 min of denaturation at 95°C; 35 cycles of 95°C for 30 s, 52°C for 30 s, and 72°C for 60 s; and a final extension of 72°C for 7 min to generate an approximately 1,000- to 1,300-bp product.

Bacterial isolates were identified by boiling 100 µL of culture medium (95°C for 10 min) to extract DNA, which were amplified with the primer set 515F/806R according to the conditions described above to generate a 350-bp product. Single-stranded DNA was removed by incubating 2 µL of the PCR product with 2 µL of a 20-U/µL exonuclease (Sigma) and 5-U/µL Arctic phosphatase (New England Biolabs) mix at 37°C for 30 min and 20 min at 80°C. Sanger sequencing was performed at the MSU Research Technology Support Facility. The forward and reverse sequences were merged, trimmed, checked for quality in Geneious 9.1, and compared to the NCBI data through BLAST and CONSTAX2 to help identify isolates (51).

### MiSeq library preparation from roots and soils.

Genomic DNA was extracted from roots with an Omega Mag-Bind Plant DNA Plus kit (Omega Bio-Tek, USA) and from soils with the MagAttract PowerSoil DNA KF (Qiagen, USA) on a KingFisher Flex purification system platform according to the manufacturer’s instructions. Each 96-well plate included 6 negative-control blank extractions, where no DNA was added, for a total of 24 negative controls. In addition, the ZymoBIOMICS Microbial Community DNA Standard D6305 (Zymo Research, USA) was used as a positive-control mock community for 16S rRNA gene libraries, and synthetic ITS sequences (52) were used as a positive-control mock community for the ITS libraries. In total, eight mock samples were included alongside the ITS or 16S libraries. Extracted DNA was quantified with a Quant-iT PicoGreen dsDNA assay kit (Thermo Fisher Scientific, USA), and the DNA concentration was normalized with an Arise Biotech EzMate601 automated pipetting system before amplification. DNAs were amplified with DreamTaq Green DNA polymerase (Thermo Scientific) for ITS library construction and Platinum *Taq* DNA polymerase (Thermo Scientific) for 16S library construction. We utilized the primer sets ITS1F/ITS2 to target the ITS1 sublocus region of ITS (53) and 515f/806r to target the V4 region of 16S rDNA (54). A modified version of the three-step PCR described previously (55) was used as previously described (56, 57). Briefly, genomic DNA was amplified with generic primers (step 1, 10 PCR cycles) to enrich fungal rDNA template. We then amplified the PCR products with primers incorporating 1- to 6-nucleotide frameshifts to increase diversity between samples (step 2, 10 PCR cycles). Finally, we incorporated 10-nucleotide indexing barcodes and Illumina adapters to the PCR products (step 3, 15 PCR cycles).

PCR products size and concentration were determined on a QIAxcel Advanced machine with a DNA Fast Analysis kit (Qiagen). Sample libraries were then normalized with a SequalPrep normalization plate kit (Thermo Fisher Scientific) and pooled. The generated amplicon library was then concentrated at approximately 20:1 with Amicon Ultra 0.5-mL 50K filters (EMDmillipore, Germany) and purified from primer dimers with Agencourt AMPure XP magnetic beads (Beckman Coulter, USA). Multiplexed libraries were sequenced on an Illumina MiSeq analyzer using the v3 600 cycle kit (Illumina, USA) by the MSU Research Technology Support Facility.

### Bioinformatic analysis.

Raw ITS and 16S reads were quality evaluated with FastQC and merged in PEAR (58). Selected reads were demultiplexed according to sample barcodes in QIIME (59), removed from Illumina adapters and sequencing primers with Cutadapt (60), quality filtered, trimmed to equal length (61, 62), dereplicated, removed from singleton sequences, and clustered into operational taxonomic units (OTUs) based on 97% similarity with UPARSE (63) algorithms. Taxonomy assignments were performed with CONSTAX2 (51) against the eukaryotic UNITE database (version 4-2-20) (64) for the ITS data and SILVA, version 138 (65), for the 16S rDNA. The bioinformatic code used in this study is available (https://github.com/PedroBeschoren/MSU-HPCC_AmpliconLibrary).

### Statistical analysis.

Contaminant OTUs were removed from the data sets using the R package decontam (66), and the removed OTUs are shown in Fig. S1. To evaluate the influence of switchgrass genotype and soil on alpha-diversity metrics (i.e., OTU richness and Shannon index), we used general linear models implemented in R through the *glm* function. To avoid violation of model assumptions we calculated the log(richness), removed samples that failed to sequence (i.e., outliers), and included sample read number (i.e., read depth) in the model, as shown previously (67).

The read number represents the bias generated by variable sequencing success among samples that always occurs during high-throughput sequencing. In some situations, a higher sequencing depth does not translate into higher biodiversity; therefore, including the read number as the first predictor in the model directly accounts for this sequencing bias, and it does not discard data the way that common practices such as rarefaction do (68).

For beta-diversity we studied two components: (i) community structure, defined as the difference in multivariate space between samples and sample groups, and (ii) community dispersion, defined as multivariate variance within each sample group. We used principal coordinate analysis (PCoA; i.e., metric multidimensional scaling) implemented in R through the *cmdscale* function combined with *adonis* (permutational multivariate analysis of variance using distance matrices) and *betadisper* (multivariate homogeneity of groups dispersions) in the vegan package (69). Differences in multivariate dispersion were tested using permutational analysis of variance (ANOVA; perm. 9999), and *P* values were corrected according to the Benjamini and Hochberg (BH) correction method (70). To model the effect of the microbial communities, as well as plant ecotype, genotype, soil origin, and soil chemistry on aerial dry plant biomass, and to identify the most important OTUs influencing aerial dry plant biomass. We used Random Forest (RF) in the *randomForest* R package. Prior to running the RF models, we run the feature algorithm implemented in *Boruta* in the Boruta R package across all predictors. To investigate autocorrelation across predictors, we coupled the RF models with principal component analysis implemented in *prcomp* in the stats R package.

A Mantel test was used to examine correlations between complex matrices, including that of bacterial communities, fungal communities, switchgrass phenotypic traits, and soil chemical composition, to approximate the three-way interactions of plant-microbe-soil. Function *mantel* in the vegan package in R was used with Spearman correlation and 999 permutation. Switchgrass phenotypic traits at 16 weeks were used, with categorical variables, including growth stage and flowering status, converted into dummy variables. Bacterial and fungal communities, switchgrass phenotypic traits, and soil chemistry were then converted into Bray-Curtis distance matrices, and the correlations among them were tested with Spearman correlation with significant level of 0.05. Plots were generated using *ggplot2* and other *ggplot2* extension R packages (71, 72). We then fit neutral models into our OTU distributions to provided information about deterministically recruited community members (73, 74), as implemented in the *tyRa* R package (75). Following the random forest modeling, the important microbial predictors selected were then passed into a linear regression model to quantitatively associate them with plant biomass. The linear models were built using backward selection with the stepAIC function in the *MASS* R package (76). In addition, we assessed the degree of correlation and significance (*P* ≤ 0.05 after BH correction) of each OTU to a target soil in relation to other soil using the function *multipatt* with the “r.g” parameter in the indicspecies R package (77), and we overlapped this information with the most important features for classification obtained with the RF models. All figures were plotted in R using the ggplot2 R package. Minimal graphical adjustments to improve figure visibility were performed using Inkscape (78).

### Data availability.

Original, demultiplexed, sequence data reads are available in the Sequence Read Archive (79) and searchable through using the BioProject accession PRJNA785605. Sanger sequences have been deposited to GenBank (80) under accession numbers OM106204 to OM106850, and OM445531 to OM445972. All data needed to reproduce the analyses and the developed R code are provided at https://github.com/Gian77/Scientific-Papers-R-Code/tree/master/daCosta_and_Benucci_etal_2021_SwithgrassMicrobiome.

## References

[1] Vellend M. 2010. Conceptual synthesis in community ecology. Q Rev Biol 85:183–206. doi:10.1086/652373. 20565040

[2] Trivedi P, Leach JE, Tringe SG, Sa T, Singh BK. 2020. Plant-microbiome interactions: from community assembly to plant health. Nat Rev Microbiol 18:607–621. doi:10.1038/s41579-020-0412-1. 32788714

[3] Berendsen RL, Pieterse CMJ, Bakker PAHM. 2012. The rhizosphere microbiome and plant health. Trends Plant Sci 17:478–486. doi:10.1016/j.tplants.2012.04.001. 22564542

[4] Bulgarelli D, Schlaeppi K, Spaepen S, van Themaat EVL, Schulze-Lefert P. 2013. Structure and functions of the bacterial microbiota of plants. Annu Rev Plant Biol 64:807–838. doi:10.1146/annurev-arplant-050312-120106. 23373698

[5] Partida-Martínez LP, Heil M. 2011. The microbe-free plant: fact or artifact? Front Plant Sci 2:100. doi:10.3389/fpls.2011.00100. 22639622 PMC3355587

[6] Morris JJ. 2018. What is the hologenome concept of evolution? F1000Res 7:1664. doi:10.12688/f1000research.14385.1. PMC6198262 30410727

[7] Hacquard S, Garrido-Oter R, González A, Spaepen S, Ackermann G, Lebeis S, McHardy AC, Dangl JL, Knight R, Ley R, Schulze-Lefert P. 2015. Microbiota and host nutrition across plant and animal kingdoms. Cell Host Microbe 17:603–616. doi:10.1016/j.chom.2015.04.009. 25974302

[8] Edwards J, Johnson C, Santos-Medellín C, Lurie E, Podishetty NK, Bhatnagar S, Eisen JA, Sundaresan V. 2015. Structure, variation, and assembly of the root-associated microbiomes of rice. Proc Natl Acad Sci U S A 112doi:10.1073/pnas.1414592112. PMC4345613 25605935

[9] Simonin M, Dasilva C, Terzi V, Ngonkeu ELM, Diouf D, Kane A, Béna G, Moulin L. 2020. Influence of plant genotype and soil on the wheat rhizosphere microbiome: evidences for a core microbiome across eight African and European soils. FEMS Microbiol Ecol 96:fiaa067. doi:10.1093/femsec/fiaa067. 32275297

[10] Fitzpatrick CR, Copeland J, Wang PW, Guttman DS, Kotanen PM, Johnson MTJ. 2018. Assembly and ecological function of the root microbiome across angiosperm plant species. Proc Natl Acad Sci U S A 115:E1157–E1165. doi:10.1073/pnas.1717617115. 29358405 PMC5819437

[11] Schlaeppi K, Dombrowski N, Oter RG, Ver Loren van Themaat E, Schulze-Lefert P. 2014. Quantitative divergence of the bacterial root microbiota in *Arabidopsis thaliana* relatives. Proc Natl Acad Sci U S A 111:585–592. doi:10.1073/pnas.1321597111. 24379374 PMC3896156

[12] Wagner MR, Lundberg DS, Del Rio TG, Tringe SG, Dangl JL, Mitchell-Olds T. 2016. Host genotype and age shape the leaf and root microbiomes of a wild perennial plant. Nat Commun 7:12151. doi:10.1038/ncomms12151. 27402057 PMC4945892

[13] Brown SP, Grillo MA, Podowski JC, Heath KD. 2021. Correction to: soil origin and plant genotype structure distinct microbiome compartments in the model legume *Medicago truncatula*. Microbiome 9:105. doi:10.1186/s40168-021-01080-3. 33971961 PMC8112050

[14] Morella NM, Weng FC-H, Joubert PM, Metcalf CJE, Lindow S, Koskella B. 2020. Successive passaging of a plant-associated microbiome reveals robust habitat and host genotype-dependent selection. Proc Natl Acad Sci U S A 117:1148–1159. doi:10.1073/pnas.1908600116. 31806755 PMC6969547

[15] Cregger MA, Veach AM, Yang ZK, Crouch MJ, Vilgalys R, Tuskan GA, Schadt CW. 2018. The Populus holobiont: dissecting the effects of plant niches and genotype on the microbiome. Microbiome 6:31. doi:10.1186/s40168-018-0413-8. 29433554 PMC5810025

[16] Longley R, Noel ZA, Benucci GMN, Chilvers MI, Trail F, Bonito G. 2020. Crop management impacts the soybean (*Glycine max*) microbiome. Front Microbiol 11:1116. doi:10.3389/fmicb.2020.01116. 32582080 PMC7283522

[17] Berg G, Smalla K. 2009. Plant species and soil type cooperatively shape the structure and function of microbial communities in the rhizosphere. FEMS Microbiol Ecol 68:1–13. doi:10.1111/j.1574-6941.2009.00654.x. 19243436

[18] Knights HE, Jorrin B, Haskett TL, Poole PS. 2021. Deciphering bacterial mechanisms of root colonization. Environ Microbiol Rep 13:428–444. doi:10.1111/1758-2229.12934. 33538402 PMC8651005

[19] Cordovez V, Rotoni C, Dini-Andreote F, Oyserman B, Carrión VJ, Raaijmakers JM. 2021. Successive plant growth amplifies genotype-specific assembly of the tomato rhizosphere microbiome. Sci Total Environ 772:144825. doi:10.1016/j.scitotenv.2020.144825. 33581524

[20] Hestrin R, Lee MR, Whitaker BK, Pett-Ridge J. 2021. The switchgrass microbiome: a review of structure, function, and taxonomic distribution. Phytobiomes J 5:14–28. doi:10.1094/PBIOMES-04-20-0029-FI.

[21] Liebig MA, Schmer MR, Vogel KP, Mitchell RB. 2008. Soil carbon storage by switchgrass grown for bioenergy. Bioenerg Res 1:215–222. doi:10.1007/s12155-008-9019-5.

[22] Milano ER, Lowry DB, Juenger TE. 2016. The genetic basis of upland/lowland ecotype divergence in switchgrass (*Panicum virgatum*). G3 (Bethesda) 6:3561–3570. doi:10.1534/g3.116.032763. 27613751 PMC5100855

[23] Pérez-Jaramillo JE, Mendes R, Raaijmakers JM. 2016. Impact of plant domestication on rhizosphere microbiome assembly and functions. Plant Mol Biol 90:635–644. doi:10.1007/s11103-015-0337-7. 26085172 PMC4819786

[24] Liu F, Hewezi T, Lebeis SL, Pantalone V, Grewal PS, Staton ME. 2019. Soil indigenous microbiome and plant genotypes cooperatively modify soybean rhizosphere microbiome assembly. BMC Microbiol 19:201. doi:10.1186/s12866-019-1572-x. 31477026 PMC6720100

[25] Turner TR, James EK, Poole PS. 2013. The plant microbiome. Genome Biol 14:209. doi:10.1186/gb-2013-14-6-209. 23805896 PMC3706808

[26] Faoro H, Alves AC, Souza EM, Rigo LU, Cruz LM, Al-Janabi SM, Monteiro RA, Baura VA, Pedrosa FO. 2010. Influence of soil characteristics on the diversity of bacteria in the Southern Brazilian Atlantic Forest. Appl Environ Microbiol 76:4744–4749. doi:10.1128/AEM.03025-09. 20495051 PMC2901723

[27] Frey SD, Knorr M, Parrent JL, Simpson RT. 2004. Chronic nitrogen enrichment affects the structure and function of the soil microbial community in temperate hardwood and pine forests. Forest Ecol Management doi:10.1016/j.foreco.2004.03.018.

[28] Rousk J, Bååth E, Brookes PC, Lauber CL, Lozupone C, Gregory Caporaso J, Knight R, Fierer N. 2010. Soil bacterial and fungal communities across a pH gradient in an arable soil. ISME J 4:1340–1351. doi:10.1038/ismej.2010.58. 20445636

[29] van der Heijden MGA, Bardgett RD, van Straalen NM. 2008. The unseen majority: soil microbes as drivers of plant diversity and productivity in terrestrial ecosystems. Ecol Lett 11:296–310. doi:10.1111/j.1461-0248.2007.01139.x. 18047587

[30] Wagg C, Jansa J, Schmid B, van der Heijden MGA. 2011. Belowground biodiversity effects of plant symbionts support aboveground productivity. Ecol Lett 14:1001–1009. doi:10.1111/j.1461-0248.2011.01666.x. 21790936

[31] Chen Q-L, Ding J, Zhu Y-G, He J-Z, Hu H-W. 2020. Soil bacterial taxonomic diversity is critical to maintaining the plant productivity. Environ Int 140:105766. doi:10.1016/j.envint.2020.105766. 32371308

[32] Richardson AE, Simpson RJ. 2011. Soil microorganisms mediating phosphorus availability update on microbial phosphorus. Plant Physiol 156:989–996. doi:10.1104/pp.111.175448. 21606316 PMC3135950

[33] Richardson AE, Barea J-M, McNeill AM, Prigent-Combaret C. 2009. Acquisition of phosphorus and nitrogen in the rhizosphere and plant growth promotion by microorganisms. Plant Soil 321:305–339. doi:10.1007/s11104-009-9895-2.

[34] Smith SE, Gianinazzi-Pearson V. 1988. Physiological interactions between symbionts in vesicular-arbuscular mycorrhizal plants. Annu Rev Plant Physiol Plant Mol Biol 39:221–244. doi:10.1146/annurev.pp.39.060188.001253.

[35] Bucher M. 2007. Functional biology of plant phosphate uptake at root and mycorrhiza interfaces. New Phytol 173:11–26. doi:10.1111/j.1469-8137.2006.01935.x. 17176390

[36] Ray P, Abraham PE, Guo Y, Giannone RJ, Engle NL, Yang ZK, Jacobson D, Hettich RL, Tschaplinski TJ, Craven KD. 2019. Scavenging organic nitrogen and remodelling lipid metabolism are key survival strategies adopted by the endophytic fungi, *Serendipita vermifera* and *Serendipita bescii* to alleviate nitrogen and phosphorous starvation *in vitro*. Environ Microbiol Rep 11:548–557. doi:10.1111/1758-2229.12757. 30970176 PMC6850091

[37] Ghimire SR, Charlton ND, Craven KD. 2009. The mycorrhizal fungus, *Sebacina vermifera*, enhances seed germination and biomass production in switchgrass (*Panicum virgatum* L). Bioenerg Res 2:51–58. doi:10.1007/s12155-009-9033-2.

[38] Ghimire SR, Craven KD. 2011. Enhancement of switchgrass (*Panicum virgatum* L.) biomass production under drought conditions by the ectomycorrhizal fungus *Sebacina vermifera*. Appl Environ Microbiol 77:7063–7067. doi:10.1128/AEM.05225-11. 21841032 PMC3187112

[39] Lee MR, Hawkes CV. 2021. Widespread co‐occurrence of *Sebacinales* and arbuscular mycorrhizal fungi in switchgrass roots and soils has limited dependence on soil carbon or nutrients. Plants People Planet 3:614–626. doi:10.1002/ppp3.10181.

[40] Lombard L, Houbraken J, Decock C, Samson RA, Meijer M, Réblová M, Groenewald JZ, Crous PW. 2016. Generic hyperdiversity in *Stachybotriaceae*. Persoonia 36:156–246. doi:10.3767/003158516X691582. 27616791 PMC4988370

[41] Salem IB, Ben Salem I, Correia KC, Boughalleb N, Michereff SJ, León M, Abad-Campos P, García-Jiménez J, Armengol J. 2013. *Monosporascus eutypoides*, a cause of root rot and vine decline in Tunisia, and evidence that *M. cannonballus* and *M. eutypoides* are distinct species. Plant Dis 97:737–743. doi:10.1094/PDIS-05-12-0464-RE. 30722587

[42] Robinson AJ, Natvig DO, Chain PSG. 2020. Genomic analysis of diverse members of the fungal genus *Monosporascus* reveals novel lineages, unique genome content and a potential bacterial associate. G3 (Bethesda) 10:2573–2583. doi:10.1534/g3.120.401489. 32580939 PMC7407469

[43] Baldani JI, Reis VM, Videira SS, Boddey LH, Baldani VLD. 2014. The art of isolating nitrogen-fixing bacteria from non-leguminous plants using N-free semi-solid media: a practical guide for microbiologists. Plant Soil 384:413–431. doi:10.1007/s11104-014-2186-6.

[44] Nyström T. 2001. Not quite dead enough: on bacterial life, culturability, senescence, and death. Arch Microbiol 176:159–164. doi:10.1007/s002030100314. 11511862

[45] Pinto D, Santos MA, Chambel L. 2015. Thirty years of viable but nonculturable state research: unsolved molecular mechanisms. Crit Rev Microbiol 41:61–76. doi:10.3109/1040841X.2013.794127. 23848175

[46] Roley SS, Duncan DS, Liang D, Garoutte A, Jackson RD, Tiedje JM, Robertson GP. 2018. Associative nitrogen fixation (ANF) in switchgrass (*Panicum virgatum*) across a nitrogen input gradient. PLoS One 13:e0197320. doi:10.1371/journal.pone.0197320. 29856843 PMC5983442

[47] Roley SS, Xue C, Hamilton SK, Tiedje JM, Robertson GP. 2019. Isotopic evidence for episodic nitrogen fixation in switchgrass (*Panicum virgatum* L.). Soil Biol Biochem 129:90–98. doi:10.1016/j.soilbio.2018.11.006.

[48] Kasmerchak CS, Schaetzl R. 2018. Soils of the GLBRC marginal land experiment (MLE) sites. Zenodo. 10.5281/ZENODO.2578238.

[49] Kleczewski NM, Bauer JT, Bever JD, Clay K, Reynolds HL. 2012. A survey of endophytic fungi of switchgrass (*Panicum virgatum*) in the Midwest, and their putative roles in plant growth. Fungal Ecol 5:521–529. doi:10.1016/j.funeco.2011.12.006.

[50] Ambrosini A, Passaglia LMP. 2017. Plant growth-promoting bacteria (PGPB): isolation and screening of PGP activities. Curr Protoc Plant Biol 2:190–209. doi:10.1002/pb.20054. 31725969

[51] Liber JA, Bonito G, Benucci GMN. 2021. CONSTAX2: improved taxonomic classification of environmental DNA markers. Bioinformatics 37:3941–3943. doi:10.1093/bioinformatics/btab347. 33961008

[52] Palmer JM, Jusino MA, Banik MT, Lindner DL. 2018. Non-biological synthetic spike-in controls and the AMPtk software pipeline improve mycobiome data. PeerJ 6:e4925. doi:10.7717/peerj.4925. 29868296 PMC5978393

[53] Monard C, Gantner S, Stenlid J. 2013. Utilizing ITS1 and ITS2 to study environmental fungal diversity using pyrosequencing. FEMS Microbiol Ecol 84:165–175. doi:10.1111/1574-6941.12046. 23176677

[54] Caporaso JG, Lauber CL, Walters WA, Berg-Lyons D, Lozupone CA, Turnbaugh PJ, Fierer N, Knight R. 2011. Global patterns of 16S rRNA diversity at a depth of millions of sequences per sample. Proc Natl Acad Sci U S A 108:4516–4522. doi:10.1073/pnas.1000080107. 20534432 PMC3063599

[55] Lundberg DS, Yourstone S, Mieczkowski P, Jones CD, Dangl JL. 2013. Practical innovations for high-throughput amplicon sequencing. Nat Methods 10:999–1002. doi:10.1038/nmeth.2634. 23995388

[56] Benucci GMN, Rennick B, Bonito G. 2020. Patient propagules: do soil archives preserve the legacy of fungal and prokaryotic communities? PLoS One 15:e0237368. doi:10.1371/journal.pone.0237368. 32780777 PMC7418970

[57] Benucci GMN, Longley R, Zhang P, Zhao Q, Bonito G, Yu F. 2019. Microbial communities associated with the black morel cultivated in greenhouses. PeerJ 7:e7744. doi:10.7717/peerj.7744. 31579614 PMC6766373

[58] Zhang J, Kobert K, Flouri T, Stamatakis A. 2014. PEAR: a fast and accurate Illumina Paired-End reAd mergeR. Bioinformatics 30:614–620. doi:10.1093/bioinformatics/btt593. 24142950 PMC3933873

[59] Caporaso JG, Kuczynski J, Stombaugh J, Bittinger K, Bushman FD, Costello EK, Fierer N, Peña AG, Goodrich JK, Gordon JI, Huttley GA, Kelley ST, Knights D, Koenig JE, Ley RE, Lozupone CA, McDonald D, Muegge BD, Pirrung M, Reeder J, Sevinsky JR, Turnbaugh PJ, Walters WA, Widmann J, Yatsunenko T, Zaneveld J, Knight R. 2010. QIIME allows analysis of high-throughput community sequencing data. Nat Methods 7:335–336. doi:10.1038/nmeth.f.303. 20383131 PMC3156573

[60] Martin M. 2011. Cutadapt removes adapter sequences from high-throughput sequencing reads. Embnet J 17:10. doi:10.14806/ej.17.1.200.

[61] Edgar RC, Flyvbjerg H. 2015. Error filtering, pair assembly and error correction for next-generation sequencing reads. Bioinformatics 31:3476–3482. doi:10.1093/bioinformatics/btv401. 26139637

[62] Edgar R. 2016. UCHIME2: improved chimera prediction for amplicon sequencing. doi:10.1101/074252.

[63] Edgar RC. 2013. UPARSE: highly accurate OTU sequences from microbial amplicon reads. Nat Methods 10:996–998. doi:10.1038/nmeth.2604. 23955772

[64] UNITE Community. 2017. PlutoF biodiversity platform. 10.15156/BIO/587475. Accessed 30 September 2019.

[65] Quast C, Pruesse E, Yilmaz P, Gerken J, Schweer T, Yarza P, Peplies J, Glöckner FO. 2013. The SILVA ribosomal RNA gene database project: improved data processing and web-based tools. Nucleic Acids Res 41:D590–D596. doi:10.1093/nar/gks1219. 23193283 PMC3531112

[66] Davis NM, Proctor DM, Holmes SP, Relman DA, Callahan BJ. 2018. Simple statistical identification and removal of contaminant sequences in marker-gene and metagenomics data. Microbiome 6:226. doi:10.1186/s40168-018-0605-2. 30558668 PMC6298009

[67] Bálint M, Bartha L, O’Hara RB, Olson MS, Otte J, Pfenninger M, Robertson AL, Tiffin P, Schmitt I. 2015. Relocation, high-latitude warming and host genetic identity shape the foliar fungal microbiome of poplars. Mol Ecol 24:235–248. doi:10.1111/mec.13018. 25443313

[68] McMurdie PJ, Holmes S. 2014. Waste not, want not: why rarefying microbiome data is inadmissible. PLoS Comput Biol 10:e1003531. doi:10.1371/journal.pcbi.1003531. 24699258 PMC3974642

[69] Oksanen J, Blanchet FG, Friendly M, Kindt R, Legendre P, McGlinn D, Minchin PR, O’Hara RB, Simpson GL, Solymos P, Stevens MHH, Szoecs E, Wagner H. 2019. vegan: Community Ecology Package, R package version. https://CRAN.R-project.org/package=vegan.

[70] Benjamini Y, Hochberg Y. 1995. Controlling the false discovery rate: a practical and powerful approach to multiple testing. J R Stat Soc Ser B (Methodological) 57:289–300. 10.1111/j.2517-6161.1995.tb02031.x. doi:10.1111/j.2517-6161.1995.tb02031.x.

[71] Kassambara A. 2020. ggpubr: “ggplot2” based publication ready plots. R package version 0.4.0. https://CRAN.R-project.org/package=ggpubr.

[72] Wickham H. 2016. ggplot2: Elegant Graphics for Data Analysis. Springer-Verlag, New York, NY. https://ggplot2.tidyverse.org.

[73] Shade A, Stopnisek N. 2019. Abundance-occupancy distributions to prioritize plant core microbiome membership. Curr Opin Microbiol 49:50–58. doi:10.1016/j.mib.2019.09.008. 31715441

[74] Sloan WT, Lunn M, Woodcock S, Head IM, Nee S, Curtis TP. 2006. Quantifying the roles of immigration and chance in shaping prokaryote community structure. Environ Microbiol 8:732–740. doi:10.1111/j.1462-2920.2005.00956.x. 16584484

[75] Sprockett D. 2020. tyRa: build models for microbiome data R package, version 0.1.0. https://danielsprockett.github.io/tyRa/.

[76] Venables WN, Ripley BD. 2013. Modern applied statistics with S-PLUS. Springer Science & Business Media, New York, NY. https://play.google.com/store/books/details?id=-vbTBwAAQBAJ.

[77] De Cáceres M, Legendre P, Moretti M. 2010. Improving indicator species analysis by combining groups of sites. Oikos 119:1674–1684. doi:10.1111/j.1600-0706.2010.18334.x.

[78] Inkscape. 2020. Inkscape Project. https://inkscape.org.

[79] Leinonen R, Sugawara H, Shumway M, International Nucleotide Sequence Database Collaboration. 2011. The sequence read archive. Nucleic Acids Res 39:D19–D21. doi:10.1093/nar/gkq1019. 21062823 PMC3013647

[80] Clark K, Karsch-Mizrachi I, Lipman DJ, Ostell J, Sayers EW. 2016. GenBank. Nucleic Acids Res 44:D67–D72. doi:10.1093/nar/gkv1276. 26590407 PMC4702903

