# Supplementary figures and images for: Soil Origin and Plant Genotype Modulate Switchgrass Aboveground Productivity and Root Microbiome Assembly
Source: mBio. 2022 Apr 6;13(2):e00079-22. doi: 10.1128/mbio.00079-22 (PMC9040762; doi:10.1128/mbio.00079-22)

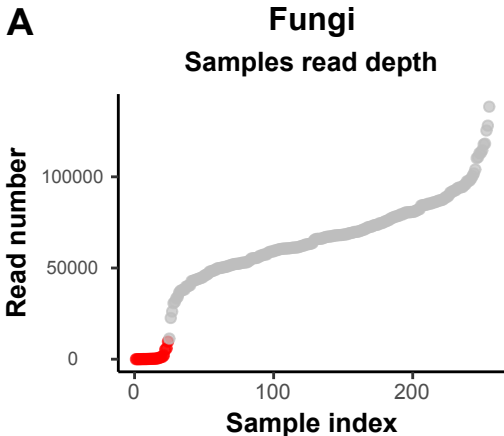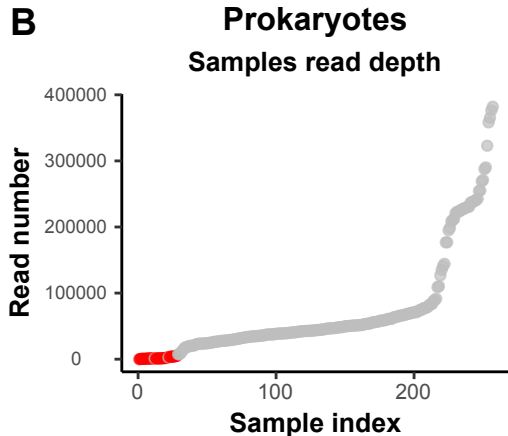

Negative control    ● FALSE    ● TRUE

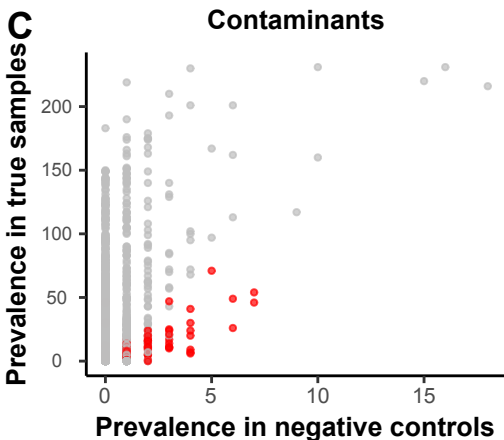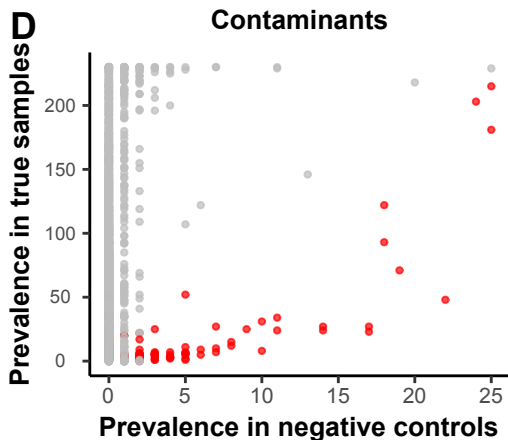

Contaminant OTUs    ● FALSE    ● TRUE

Supplement: FIG S1 [file mbio.00079-22-sf001.pdf]

# A Fungi richness

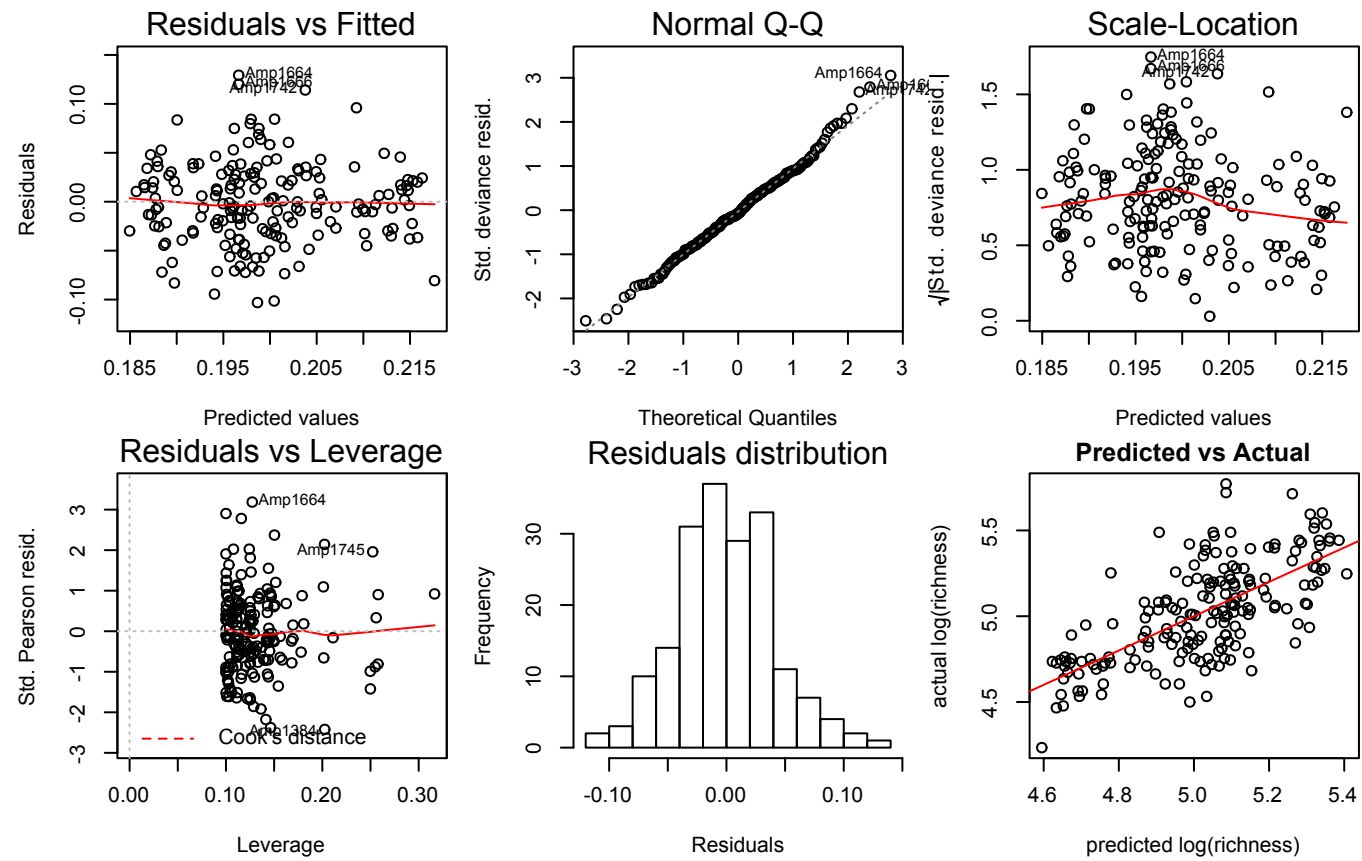

# C Fungi Shannon index

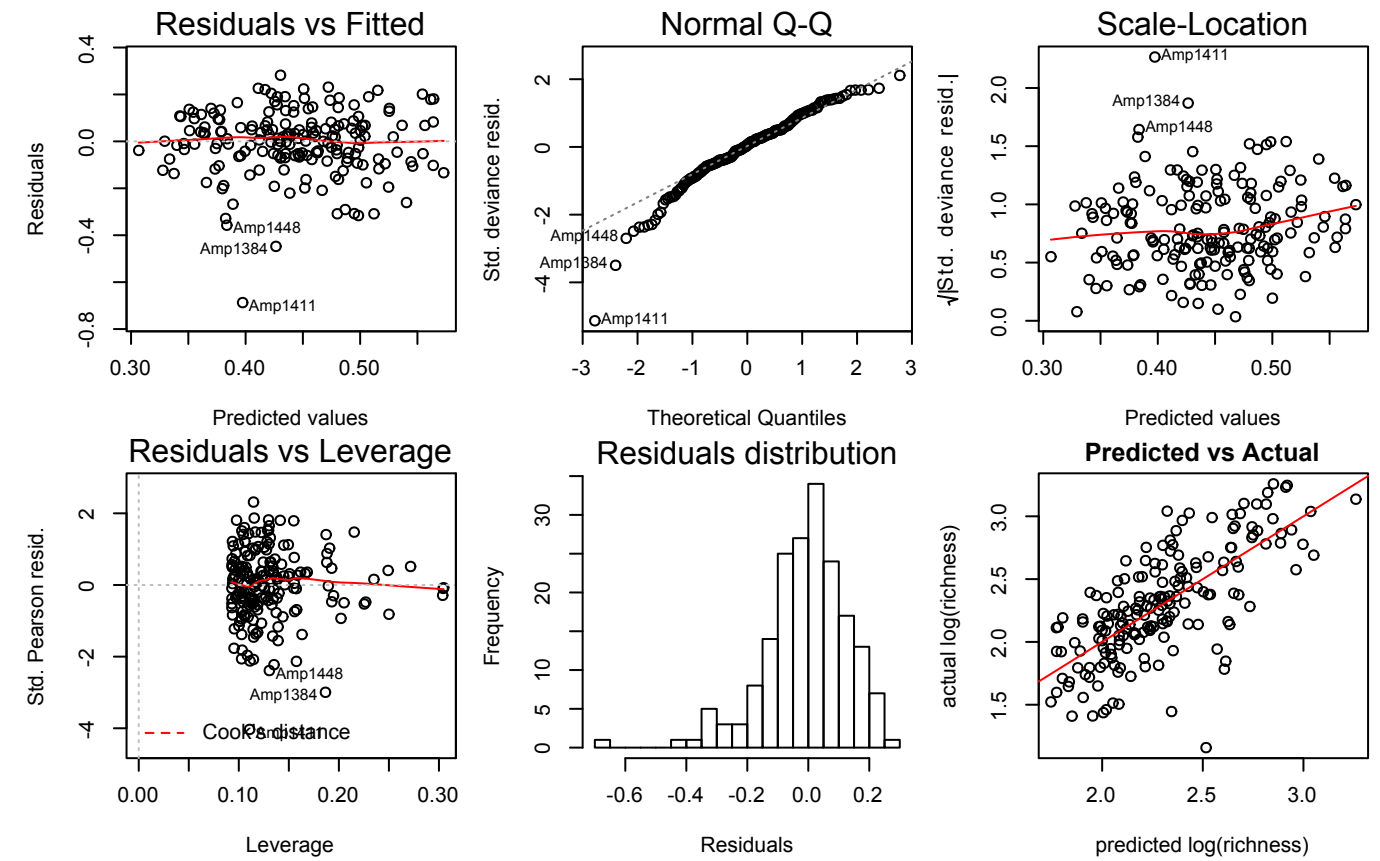

# B Bacteria richness

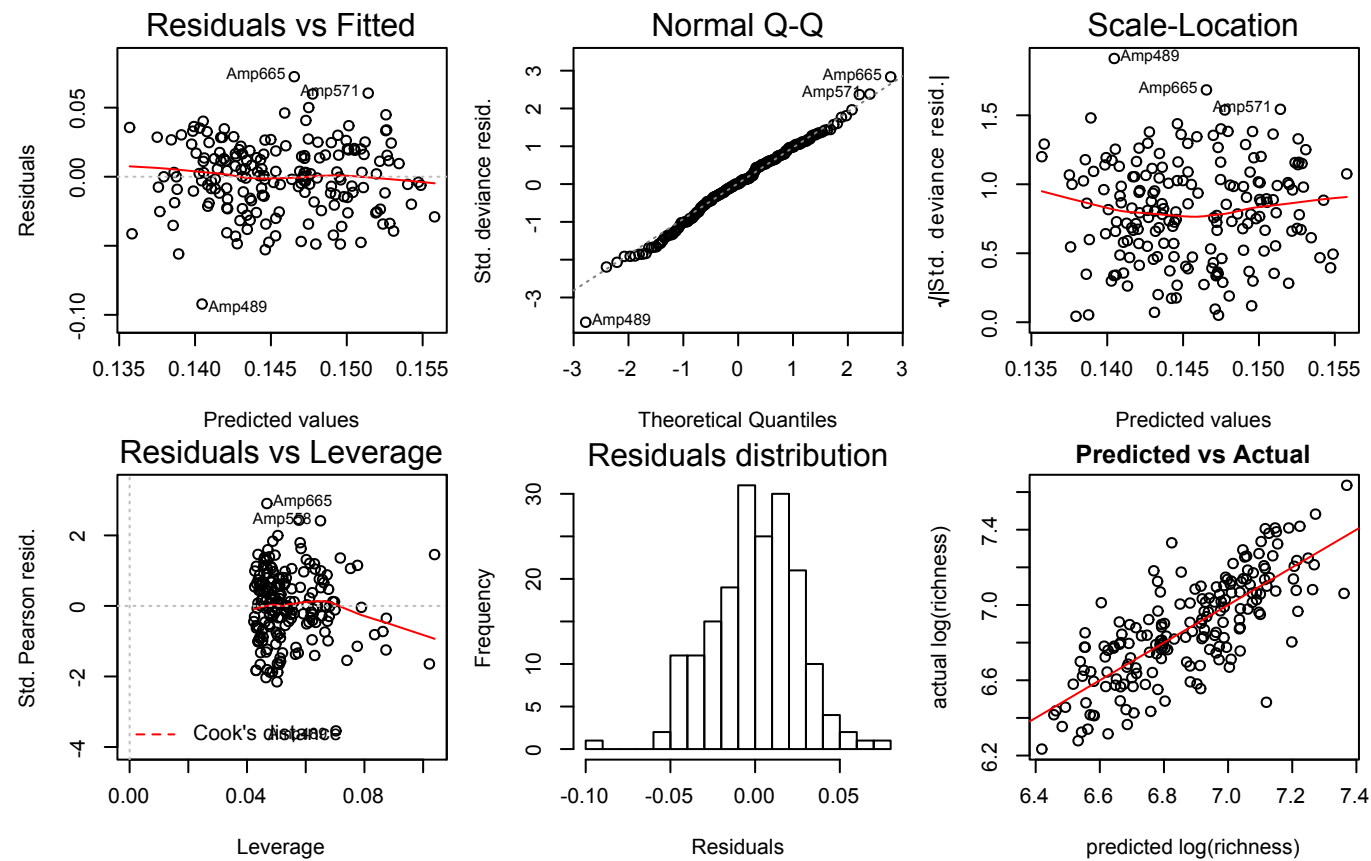

# D Bacteria Shannon index

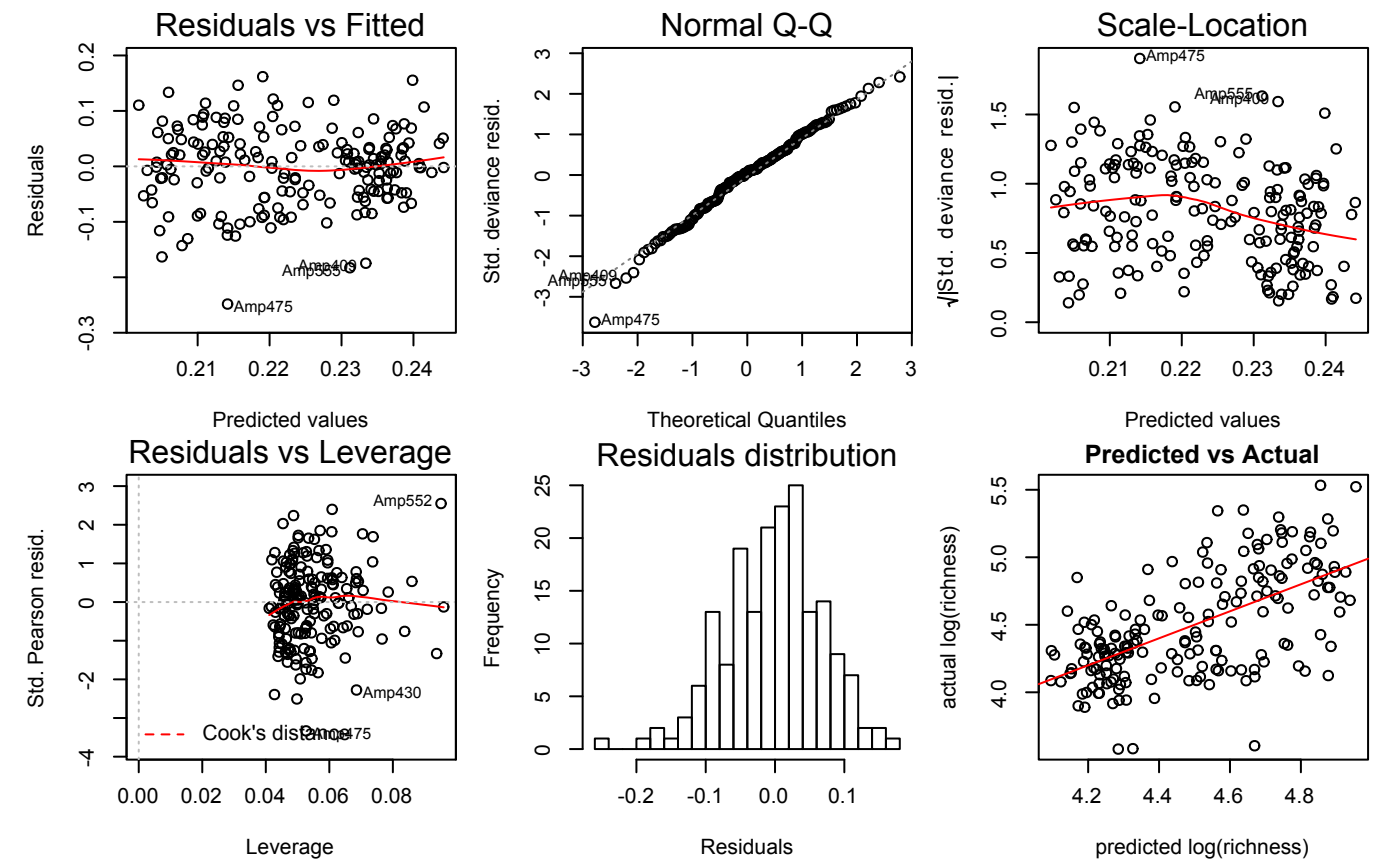

Supplement: FIG S2 [file mbio.00079-22-sf002.pdf]

### Fungi soil

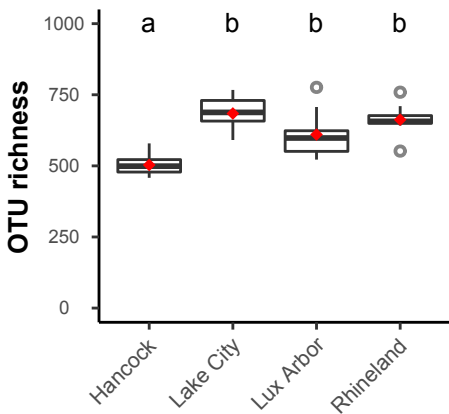

### Fungi roots

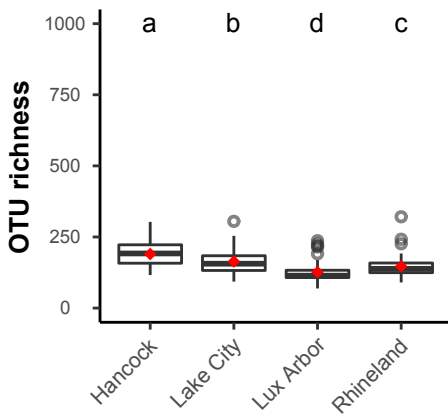

### Bacteria soil

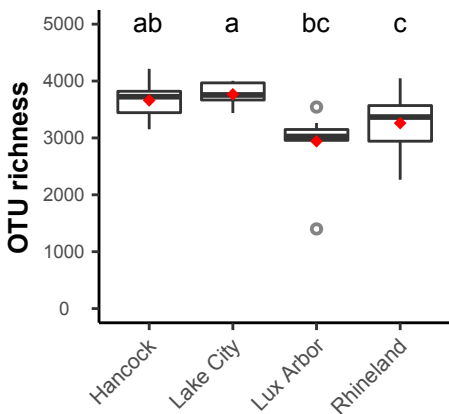

### Bacteria roots

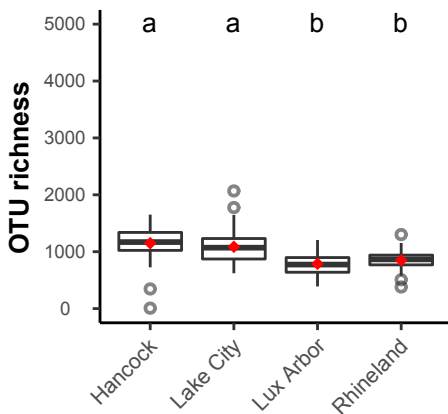

Supplement: FIG S3 [file mbio.00079-22-sf003.pdf]

A

Fungi

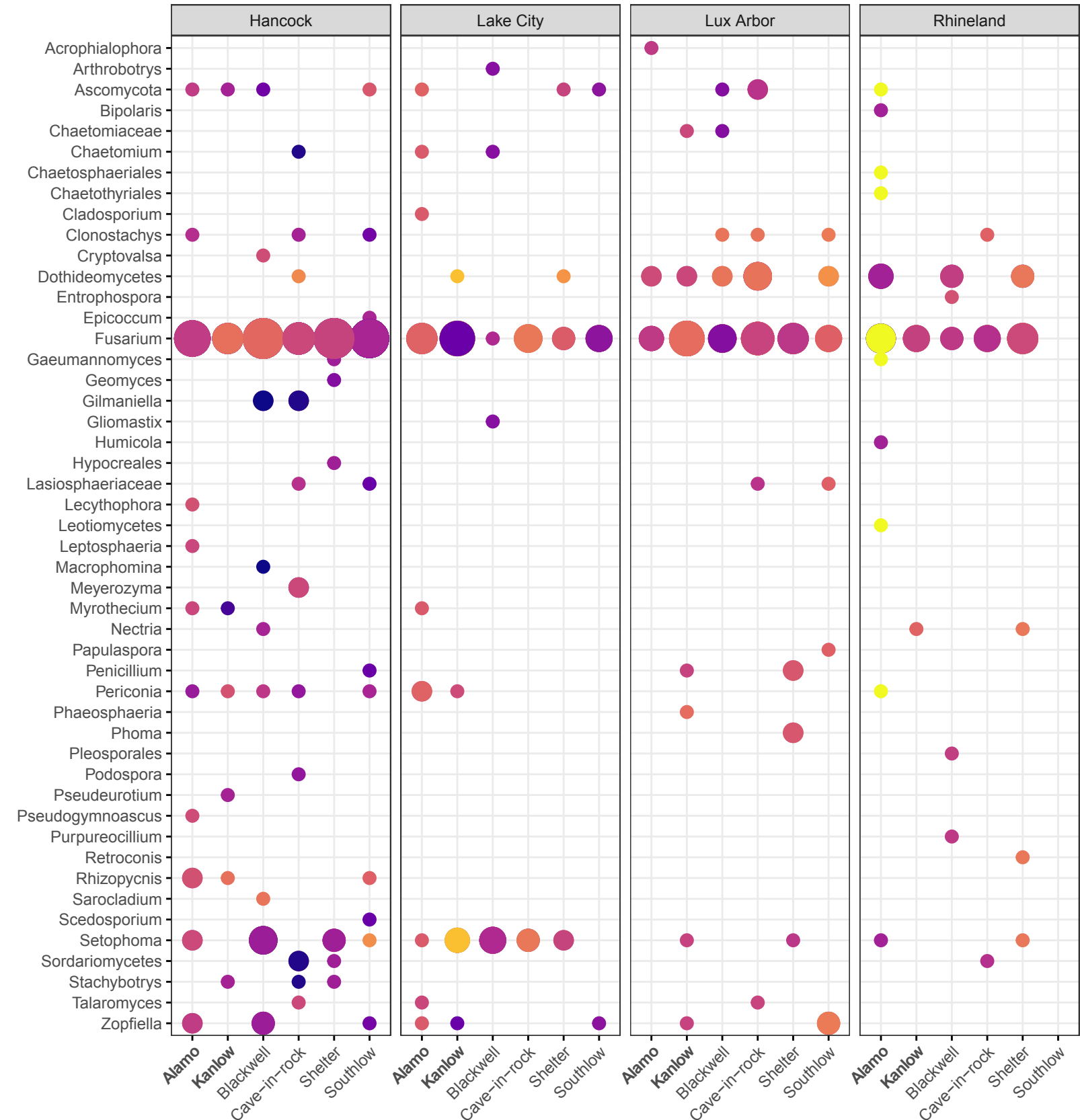

B

Bacteria

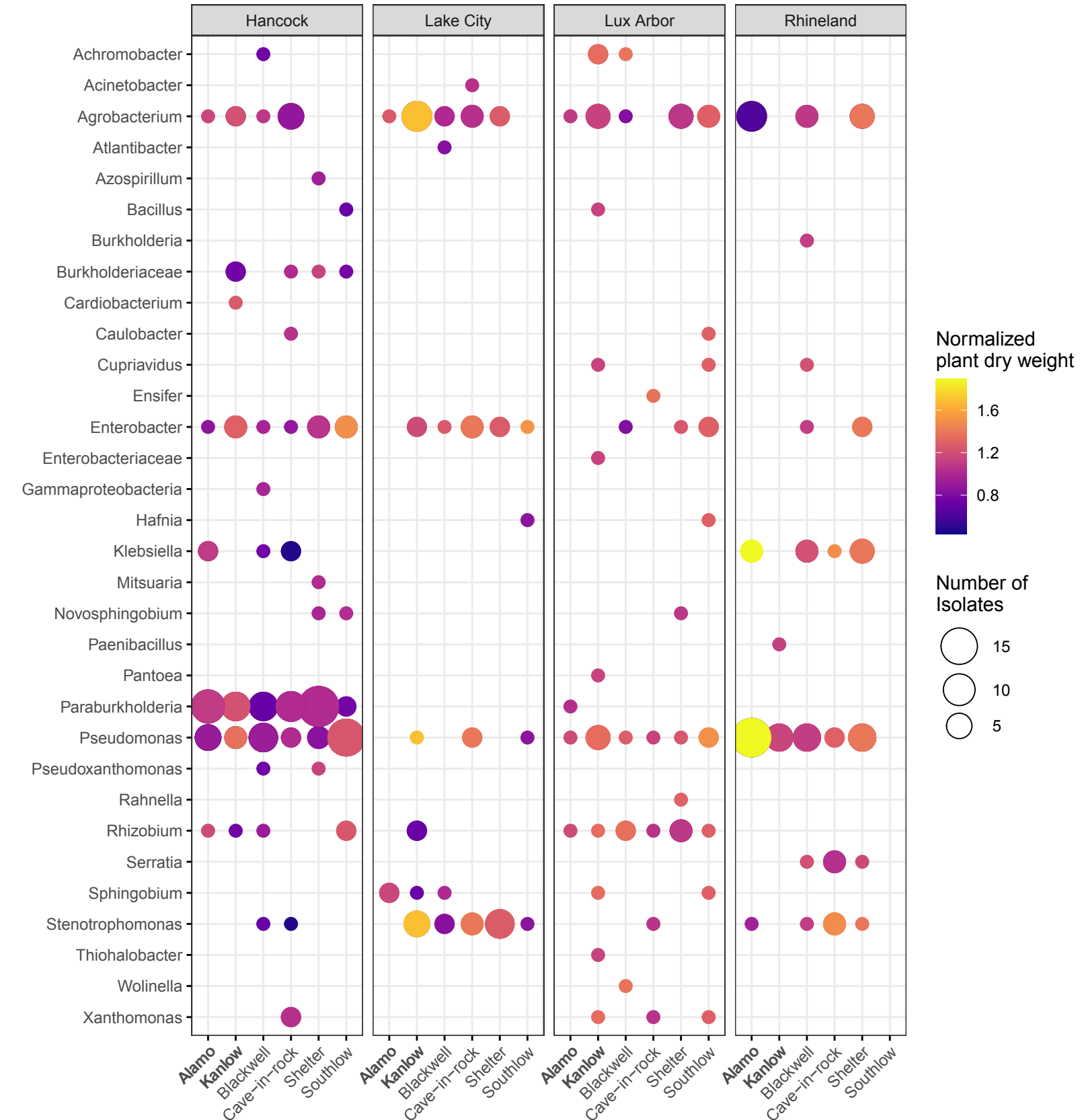

C

Fungi

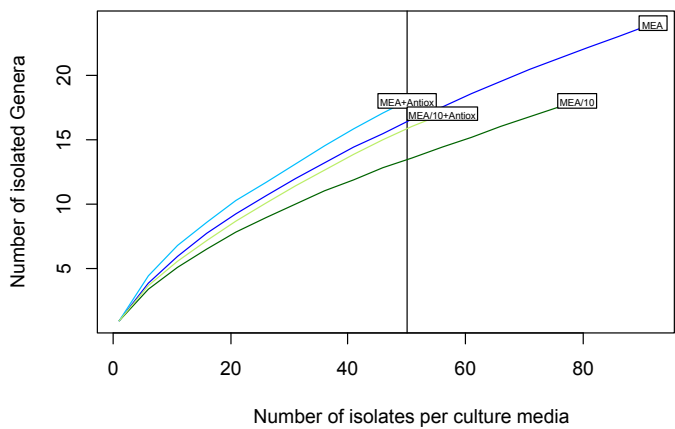

D

Bacteria

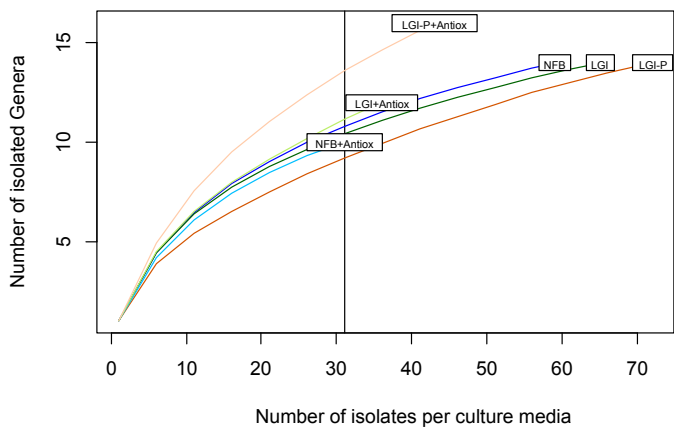

Supplement: FIG S5 [file mbio.00079-22-sf005.pdf]
