# Supplementary material for: Soil Origin and Plant Genotype Modulate Switchgrass Aboveground Productivity and Root Microbiome Assembly
Source: mBio. 2022 Apr 6;13(2):e00079-22. doi: 10.1128/mbio.00079-22 (PMC9040762; doi:10.1128/mbio.00079-22)
Supplement: TABLE S1 [file mbio.00079-22-st001.pdf]

**Table S1.** Generalized linear models on alpha diversity metrics.

We used `glm()` to model the effect of genotype and soil on alpha diversity metrics (*log* of OTU richness and Shannon index). We added read number as the first factor in the model to account for variation in read depth across samples before all the other factors. To select for best model we used a step forward approach adding a factor and testing for model improvement. All models were finally compared using `compareGLM()` and the most parsimonious model was selected according to the lowest $AIC_c$ (or *BIC* when close $AIC_c$ values) and comparison with other nested models using `anova()` with `test="Chisq"`. Significance of different model predictors was assessed using `Anova()` in the *car* R package and type II test. Diagnostic plots for the best models are reported in Fig. S2.

## Anova tables

Fungal communities:

```
Analysis of Deviance Table (Type II tests)

Response: log(richness)
               Sum Sq  Df F value                Pr(>F)
readNo        0.00567   1  2.7583              0.098719 .
Soil          0.18081   3 29.3393 0.0000000000000003755 ***
Genotype      0.02327   5  2.2660              0.050457 .
Soil:Genotype 0.08050  15  2.6125              0.001536 **
Residuals     0.32662 159
---
Signif. codes:  0 '***' 0.001 '**' 0.01 '*' 0.05 '.' 0.1 ' ' 1

Analysis of Deviance Table (Type II tests)

Response: shannon
          Sum Sq  Df F value            Pr(>F)
readNo    0.9678   1 46.0173 0.000000000176187 ***
Genotype  0.0586   5  0.5576            0.7324
Soil      1.3627   3 21.5966 0.000000000006134 ***
Residuals 3.6596 174
---
Signif. codes:  0 '***' 0.001 '**' 0.01 '*' 0.05 '.' 0.1 ' ' 1
```

Bacterial communities:

```
Analysis of Deviance Table (Type II tests)

Response: log(richness)
            Sum Sq  Df F value               Pr(>F)
readNo    0.067487   1 97.3459 <0.0000000000000002 ***
Genotype  0.004347   5  1.2541                0.286
Soil      0.151812   3 72.9935 <0.0000000000000002 ***
Residuals 0.120628 174
---
```

```
Signif. codes:  0 '***' 0.001 '**' 0.01 '*' 0.05 '.' 0.1 ' ' 1

Analysis of Deviance Table (Type II tests)

Response: shannon
           Sum Sq  Df F value           Pr(>F)    
readNo    0.01362   1  2.6223           0.1072    
Genotype  0.03715   5  1.4299           0.2157    
Soil      0.38502   3 24.7021 0.00000000000023 ***
Residuals 0.90403 174                             
---
Signif. codes:  0 '***' 0.001 '**' 0.01 '*' 0.05 '.' 0.1 ' ' 1
```

## Normality of models residuals

### Fungal communities log(richness)

```
     Shapiro-Wilk normality test

data:  fit_fungi_rich_m4$residuals
W = 0.98906, p-value = 0.1695
```

### Fungal communities Shannon index

```
     Shapiro-Wilk normality test

data:  fit_fungi_shan_m3$residuals
W = 0.98542, p-value = 0.05318
```

### Bacterial communities log(richness)

```
     Shapiro-Wilk normality test

data:  fit_bact_rich_m3$residuals
W = 0.99271, p-value = 0.4895
```

### Bacterial communities Shannon index

```
     Shapiro-Wilk normality test

data:  fit_bact_shan_m3$residuals
W = 0.99442, p-value = 0.7215
```
