# Supplementary material for: Soil Origin and Plant Genotype Modulate Switchgrass Aboveground Productivity and Root Microbiome Assembly
Source: mBio. 2022 Apr 6;13(2):e00079-22. doi: 10.1128/mbio.00079-22 (PMC9040762; doi:10.1128/mbio.00079-22)
Supplement: TABLE S2 [file mbio.00079-22-st002.pdf]

Table S2. Permutational Multivariate Analysis of Variance Using Distance Matrices [`adonis()`]. Models were fitted using ungrouped and grouped soil samples. Soil were grouped according to high or low beta dispersion [see `betadisper()`].

`adonis()` twice for each community inverting the order of the factors in the model because of the unbalanced design of the study. Read No is the number of reads obtained in each sample from sequencing (i.e. depth), and represents a sequencing bias. We added read number as the first factor in the model to account for variation in read depth across samples before all the other factors (see manuscript text M&M section). In grouped soils we also run `adonis()` using the strata option to constrain the permutation within soils. `betadisper()` analysis followed by `anova()` was used to assess significant differences in homogeneity of multivariate dispersions (distance from sample centroids) across sample groups.

A) Ungrouped soil samples

First adonis model for fungal communities:

```
adonis(formula = otu_fungi ~ readNo + Genotype * Soil, data =
meta_fungi, permutations = 9999, method = "bray")

Permutation: free
Number of permutations: 9999

Terms added sequentially (first to last)

               Df SumsOfSqs MeanSqs F.Model      R2 Pr(>F)    
readNo          1     3.459  3.4589  28.542 0.07690 0.0001 ***
Genotype        5     1.246  0.2493   2.057 0.02771 0.0004 ***
Soil            3    19.066  6.3553  52.443 0.42389 0.0001 ***
Genotype:Soil  15     1.938  0.1292   1.066 0.04310 0.2911    
Residuals     159    19.269  0.1212         0.42840           
Total         183    44.978                 1.00000           
---
Signif. codes:  0 '***' 0.001 '**' 0.01 '*' 0.05 '.' 0.1 ' ' 1
```

Second adonis model for fungal communities:

```
adonis(formula = otu_fungi ~ readNo + Soil * Genotype, data =
meta_fungi, permutations = 9999, method = "bray")

Permutation: free
Number of permutations: 9999

Terms added sequentially (first to last)

               Df SumsOfSqs MeanSqs F.Model      R2 Pr(>F)    
readNo          1     3.459  3.4589  28.542 0.07690 0.0001 ***
Soil            3    19.456  6.4852  53.515 0.43256 0.0001 ***
Genotype        5     0.857  0.1713   1.414 0.01905 0.0509 .
```

```
Soil:Genotype  15     1.938  0.1292   1.066 0.04310 0.2925
Residuals     159    19.269  0.1212         0.42840
Total         183    44.978                 1.00000
---
Signif. codes:  0 '***' 0.001 '**' 0.01 '*' 0.05 '.' 0.1 ' ' 1
```

First adonis model for bacterial communities:

```
Call:
adonis(formula = otu_bact ~ readNo + Genotype * Soil, data =
meta_bact, permutations = 9999, method = "bray")

Permutation: free
Number of permutations: 9999

Terms added sequentially (first to last)

               Df SumsOfSqs MeanSqs F.Model      R2 Pr(>F)
readNo          1     4.667  4.6667  43.650 0.12850 0.0001 ***
Genotype        5     1.245  0.2490   2.329 0.03428 0.0001 ***
Soil            3    11.766  3.9221  36.686 0.32399 0.0001 ***
Genotype:Soil  15     1.641  0.1094   1.023 0.04517 0.3944
Residuals     159    16.999  0.1069         0.46806
Total         183    36.318                 1.00000
---
Signif. codes:  0 '***' 0.001 '**' 0.01 '*' 0.05 '.' 0.1 ' ' 1
```

Second adonis model for bacterial communities:

```
Call:
adonis(formula = otu_bact ~ readNo + Soil * Genotype, data =
meta_bact, permutations = 9999, method = "bray")

Permutation: free
Number of permutations: 9999

Terms added sequentially (first to last)

               Df SumsOfSqs MeanSqs F.Model      R2 Pr(>F)
readNo          1     4.667  4.6667  43.650 0.12850 0.0001 ***
Soil            3    12.091  4.0302  37.697 0.33291 0.0001 ***
Genotype        5     0.921  0.1842   1.723 0.02535 0.0029 **
Soil:Genotype  15     1.641  0.1094   1.023 0.04517 0.3926
Residuals     159    16.999  0.1069         0.46806
Total         183    36.318                 1.00000
---
Signif. codes:  0 '***' 0.001 '**' 0.01 '*' 0.05 '.' 0.1 ' ' 1
```

Fungi betadisper soil:

```
Call: anova(betadisper(d = vegdist(otu_fungi, method = "bray"), group
= meta_fungi$Soil), permutations = 9999)

Analysis of Variance Table

Response: Distances
           Df  Sum Sq  Mean Sq F value    Pr(>F)    
Groups      3 0.28238 0.094127  10.052 3.709e-06 ***
Residuals 180 1.68555 0.009364                      
---
Signif. codes:  0 '***' 0.001 '**' 0.01 '*' 0.05 '.' 0.1 ' ' 1
```

Fungi betadisper genotype:

```
Call: anova(betadisper(d = vegdist(otu_fungi, method = "bray"), group
= meta_fungi$Genotype), permutations = 9999)

Analysis of Variance Table

Response: Distances
           Df  Sum Sq   Mean Sq F value Pr(>F)
Groups      5 0.03771 0.0075420  1.0177 0.4087
Residuals 178 1.31910 0.0074107
```

Bacteria betadisper soil:

```
Call: anova(betadisper(d = vegdist(otu_bact, method = "bray"), group
= meta_bact$Soil), permutations = 9999)

Analysis of Variance Table

Response: Distances
           Df  Sum Sq   Mean Sq F value   Pr(>F)   
Groups      3 0.08233 0.0274430  5.5327 0.001177 **
Residuals 180 0.89283 0.0049602                    
---
Signif. codes:  0 '***' 0.001 '**' 0.01 '*' 0.05 '.' 0.1 ' ' 1
```

Bacteria betadisper genotype:

```
Call: anova(betadisper(d = vegdist(otu_bact, method = "bray"), group
= meta_bact$Genotype), permutations = 9999)

Analysis of Variance Table

Response: Distances
           Df  Sum Sq   Mean Sq F value Pr(>F)
Groups      5 0.02456 0.0049113  1.4023 0.2256
```

```
Residuals 178 0.62343 0.0035024
```

B) Grouped soil samples

First adonis model for fungal communities high diversity:

```
Call: adonis(formula =
phyloseq::distance(t(otu_table(physeq_fungi_new_H)),      method =
"bray") ~ LibrarySize + Genotype * Soil_location,      data =
metadata_fungi_H, permutations = 999)

Permutation: free
Number of permutations: 999

Terms added sequentially (first to last)

                        Df SumsOfSqs MeanSqs F.Model      R2 Pr(>F)
LibrarySize              1    1.8751  1.8751  13.788 0.08406  0.001
***
Genotype                 5    1.1043  0.2209   1.624 0.04951  0.014 *
Soil_location            1    5.8045  5.8045  42.683 0.26023  0.001
***
Genotype:Soil_location   5    0.8742  0.1748   1.286 0.03919 0.128
Residuals               93   12.6469  0.1360         0.56700
Total                  105   22.3050                 1.00000
---
Signif. codes:  0 '***' 0.001 '**' 0.01 '*' 0.05 '.' 0.1 ' ' 1
```

Second adonis model for fungal communities high diversity:

```
Call: adonis(formula =
phyloseq::distance(t(otu_table(physeq_fungi_new_H)),      method =
"bray") ~ LibrarySize + Soil_location * Genotype,      data =
metadata_fungi_H, permutations = 999)

Permutation: free
Number of permutations: 999

Terms added sequentially (first to last)

                        Df SumsOfSqs MeanSqs F.Model      R2 Pr(>F)
LibrarySize              1    1.8751  1.8751  13.788 0.08406  0.001
***
Soil_location            1    5.8370  5.8370  42.923 0.26169  0.001
***
Genotype                 5    1.0718  0.2144   1.576 0.04805  0.022 *
Soil_location:Genotype   5    0.8742  0.1748   1.286 0.03919  0.123
Residuals               93   12.6469  0.1360         0.56700
Total                  105   22.3050                 1.00000
```

```
---
Signif. codes:  0 '***' 0.001 '**' 0.01 '*' 0.05 '.' 0.1 ' ' 1
```

Third adonis model for fungal communities high diversity:

```
Call: adonis(formula =
phyloseq::distance(t(otu_table(physeq_fungi_new_H)),      method =
"bray") ~ LibrarySize + Genotype, data = metadata_fungi_H,
permutations = 999, strata = metadata_fungi_H$Soil_location)

Blocks:  strata
Permutation: free
Number of permutations: 999

Terms added sequentially (first to last)

             Df SumsOfSqs MeanSqs F.Model      R2 Pr(>F)
LibrarySize   1    1.8751 1.87507  9.6055 0.08406  0.001 ***
Genotype      5    1.1043 0.22087  1.1315 0.04951  0.033 *
Residuals    99   19.3256 0.19521         0.86642
Total       105   22.3050                 1.00000
---
Signif. codes:  0 '***' 0.001 '**' 0.01 '*' 0.05 '.' 0.1 ' ' 1
```

First adonis model for fungal communities low diversity:

```
Call: adonis(formula =
phyloseq::distance(t(otu_table(physeq_fungi_new_L)),      method =
"bray") ~ LibrarySize + Genotype * Soil_location,      data =
metadata_fungi_L, permutations = 999)

Permutation: free
Number of permutations: 999

Terms added sequentially (first to last)

                       Df SumsOfSqs MeanSqs F.Model      R2 Pr(>F)
LibrarySize             1    1.2804  1.2804  12.163 0.09096  0.001
***
Genotype                5    0.8888  0.1778   1.689 0.06314  0.022 *
Soil_location           1    3.9559  3.9559  37.578 0.28103  0.001
***
Genotype:Soil_location  5    0.4772  0.0954   0.907 0.03390  0.609
Residuals              71    7.4742  0.1053         0.53097
Total                  83   14.0765                 1.00000
---
Signif. codes:  0 '***' 0.001 '**' 0.01 '*' 0.05 '.' 0.1 ' ' 1
```

Second adonis model for fungal communities low diversity:

```
Call: adonis(formula = 
phyloseq::distance(t(otu_table(physeq_fungi_new_L)),      method = 
"bray") ~ LibrarySize + Soil_location * Genotype,      data = 
metadata_fungi_L, permutations = 999) 

Permutation: free
Number of permutations: 999

Terms added sequentially (first to last)

                       Df SumsOfSqs MeanSqs F.Model      R2 Pr(>F)    
LibrarySize             1    1.2804  1.2804  12.163 0.09096  0.001 
***
Soil_location           1    4.1986  4.1986  39.884 0.29827  0.001 
***
Genotype                5    0.6460  0.1292   1.227 0.04589  0.189    
Soil_location:Genotype  5    0.4772  0.0954   0.907 0.03390  0.627    
Residuals              71    7.4742  0.1053         0.53097           
Total                  83   14.0765                 1.00000           
---
Signif. codes:  0 ‘***’ 0.001 ‘**’ 0.01 ‘*’ 0.05 ‘.’ 0.1 ‘ ’ 1
```

Third adonis model for fungal communities low diversity:

```
Call: adonis(formula = 
phyloseq::distance(t(otu_table(physeq_fungi_new_L)),      method = 
"bray") ~ LibrarySize + Genotype, data = metadata_fungi_L, 
permutations = 999, strata = metadata_fungi_L$Soil_location) 

Blocks:  strata 
Permutation: free
Number of permutations: 999

Terms added sequentially (first to last)

            Df SumsOfSqs MeanSqs F.Model      R2 Pr(>F)    
LibrarySize  1    1.2804 1.28039  8.2798 0.09096  0.001 ***
Genotype     5    0.8888 0.17776  1.1495 0.06314  0.089 .  
Residuals   77   11.9073 0.15464         0.84590           
Total       83   14.0765                 1.00000           
---
Signif. codes:  0 ‘***’ 0.001 ‘**’ 0.01 ‘*’ 0.05 ‘.’ 0.1 ‘ ’ 1
```

First adonis model for bacterial communities high diversity:

```
Call: adonis(formula = 
phyloseq::distance(t(otu_table(physeq_bact_new_H)),      method = 
```

```
"bray") ~ LibrarySize + Genotype * Soil_location,      data =
metadata_bact_H, permutations = 999)

Permutation: free
Number of permutations: 999

Terms added sequentially (first to last)

                        Df SumsOfSqs MeanSqs F.Model      R2 Pr(>F)
LibrarySize              1    3.0909  3.0909 23.7081 0.15266  0.001
***
Genotype                 5    1.0527  0.2105  1.6150 0.05200  0.007
**
Soil_location            1    3.2303  3.2303 24.7777 0.15955  0.001
***
Genotype:Soil_location   5    0.7481  0.1496  1.1477 0.03695  0.206
Residuals               93   12.1246  0.1304         0.59884
Total                  105   20.2467                 1.00000
---
Signif. codes:  0 '***' 0.001 '**' 0.01 '*' 0.05 '.' 0.1 ' ' 1
```

Second adonis model for bacterial communities high diversity:

```
Call: adonis(formula =
phyloseq::distance(t(otu_table(physeq_bact_new_H)),      method =
"bray") ~ LibrarySize + Soil_location * Genotype,      data =
metadata_bact_H, permutations = 999)

Permutation: free
Number of permutations: 999

Terms added sequentially (first to last)

                        Df SumsOfSqs MeanSqs F.Model      R2 Pr(>F)
LibrarySize              1    3.0909  3.0909 23.7081 0.15266  0.001
***
Soil_location            1    3.3864  3.3864 25.9748 0.16726  0.001
***
Genotype                 5    0.8967  0.1793  1.3755 0.04429  0.042 *
Soil_location:Genotype   5    0.7481  0.1496  1.1477 0.03695  0.228
Residuals               93   12.1246  0.1304         0.59884
Total                  105   20.2467                 1.00000
---
Signif. codes:  0 '***' 0.001 '**' 0.01 '*' 0.05 '.' 0.1 ' ' 1
```

Third adonis model for bacterial communities high diversity:

```
Call: adonis(formula =
phyloseq::distance(t(otu_table(physeq_bact_new_H)),      method =
```

```
"bray") ~ LibrarySize + Genotype, data = metadata_bact_H,
permutations = 999, strata = metadata_bact_H$Soil_location)

Blocks:  strata
Permutation: free
Number of permutations: 999

Terms added sequentially (first to last)

             Df SumsOfSqs MeanSqs F.Model      R2 Pr(>F)
LibrarySize   1    3.0909 3.09088 19.0024 0.15266  0.001 ***
Genotype      5    1.0527 0.21055  1.2944 0.05200  0.016 *
Residuals    99   16.1031 0.16266         0.79534
Total       105   20.2467                 1.00000
---
Signif. codes:  0 '***' 0.001 '**' 0.01 '*' 0.05 '.' 0.1 ' ' 1
```

First adonis model for bacterial communities low diversity:

```
Call: adonis(formula =
phyloseq::distance(t(otu_table(physeq_bact_new_L)),      method =
"bray") ~ LibrarySize + Genotype * Soil_location,      data =
metadata_bact_L, permutations = 999)

Permutation: free
Number of permutations: 999

Terms added sequentially (first to last)

                       Df SumsOfSqs MeanSqs F.Model      R2 Pr(>F)
LibrarySize             1    2.1016 2.10159 22.5725 0.18849  0.001
***
Genotype                5    0.5710 0.11420  1.2266 0.05121  0.116
Soil_location           1    1.4459 1.44587 15.5297 0.12968  0.001
***
Genotype:Soil_location  5    0.4207 0.08415  0.9038 0.03773  0.640
Residuals              71    6.6104 0.09310         0.59288
Total                  83   11.1496                 1.00000
---
Signif. codes:  0 '***' 0.001 '**' 0.01 '*' 0.05 '.' 0.1 ' ' 1
```

Second adonis model for bacterial communities low diversity:

```
Call: adonis(formula =
phyloseq::distance(t(otu_table(physeq_bact_new_L)),      method =
"bray") ~ LibrarySize + Soil_location * Genotype,      data =
metadata_bact_L, permutations = 999)

Permutation: free
```

```
Number of permutations: 999

Terms added sequentially (first to last)

                       Df SumsOfSqs MeanSqs F.Model      R2 Pr(>F)
LibrarySize             1    2.1016 2.10159 22.5725 0.18849  0.001
***
Soil_location           1    1.4750 1.47500 15.8425 0.13229  0.001
***
Genotype                5    0.5419 0.10838  1.1641 0.04860  0.202
Soil_location:Genotype  5    0.4207 0.08415  0.9038 0.03773  0.644
Residuals              71    6.6104 0.09310         0.59288
Total                  83   11.1496                 1.00000
---
Signif. codes:  0 '***' 0.001 '**' 0.01 '*' 0.05 '.' 0.1 ' ' 1
```

Third adonis model for bacterial communities low diversity:

```
Call: adonis(formula =
phyloseq::distance(t(otu_table(physeq_bact_new_L)),      method =
"bray") ~ LibrarySize + Genotype, data = metadata_bact_L,
permutations = 999, strata = metadata_bact_L$Soil_location)

Blocks:  strata
Permutation: free
Number of permutations: 999

Terms added sequentially (first to last)

            Df SumsOfSqs MeanSqs F.Model      R2 Pr(>F)
LibrarySize  1    2.1016 2.10159 19.0896 0.18849  0.001 ***
Genotype     5    0.5710 0.11420  1.0374 0.05121  0.279
Residuals   77    8.4770 0.11009         0.76030
Total       83   11.1496                 1.00000
---
Signif. codes:  0 '***' 0.001 '**' 0.01 '*' 0.05 '.' 0.1 ' ' 1
```

Fungi betadisper high diversity:

```
> anova(
+   betadisper(phyloseq::distance(t(otu_table(physeq_fungi_new_H)),
method="bray"), metadata_fungi_H$Genotype),
+   permutations = 999)

Analysis of Variance Table

Response: Distances
           Df  Sum Sq   Mean Sq F value Pr(>F)
Groups      5 0.00985 0.0019692  0.1671 0.9741
```

```
Residuals 100 1.17835 0.0117835
```

Fungi betadisper low diversity:

```
> anova(
+   betadisper(phyloseq::distance(t(otu_table(physeq_fungi_new_L)),
method="bray"), metadata_fungi_H$Genotype),
+   permutations = 999)

Analysis of Variance Table

Response: Distances
          Df  Sum Sq   Mean Sq F value Pr(>F)
Groups     5 0.01946 0.0038915  0.4521 0.8105
Residuals 78 0.67139 0.0086076
```

Bacteria betadisper high diversity:

```
Analysis of Variance Table

> anova(
+   betadisper(phyloseq::distance(t(otu_table(physeq_bact_new_H)),
method="bray"), metadata_bact_H$Genotype),
+   permutations = 999)

Response: Distances
           Df  Sum Sq   Mean Sq F value Pr(>F)
Groups      5 0.01523 0.0030454  0.4281 0.8281
Residuals 100 0.71139 0.0071139
```

Bacteria betadisper low diversity:

```
Analysis of Variance Table

> anova(
+   betadisper(phyloseq::distance(t(otu_table(physeq_bact_new_L)),
method="bray"), metadata_bact_L$Genotype),
+   permutations = 999)

Response: Distances
          Df  Sum Sq   Mean Sq F value Pr(>F)
Groups     5 0.02167 0.0043349  0.9037 0.4831
Residuals 78 0.37417 0.0047971
```
