# Supplementary material for: Soil Origin and Plant Genotype Modulate Switchgrass Aboveground Productivity and Root Microbiome Assembly
Source: mBio. 2022 Apr 6;13(2):e00079-22. doi: 10.1128/mbio.00079-22 (PMC9040762; doi:10.1128/mbio.00079-22)
Supplement: TABLE S3 [file mbio.00079-22-st003.pdf]

Table S3. A) Fungal and bacterial Random Forest predictors (OTUs) of plant biomass. B) Linear relationship between selected OTUs and aerial plant biomass.

A) Random Forest predictors. ID = Unique identifier for each OTU, % increase MSE (mean squared error), Increase in Node Purity, Kingdom, OTU Taxonomy, Isolate match (Percentage similarity between OTU and strain sequences); Isolate percent id (% identity of BLAST between OTU and strain reference sequences); Isolate_query_coverage (query coverage of BLAST between OTU and strain reference sequence), glbrc (presence in the references database), model_fit_class (fit class of OTUs in neutral model predictions: above, below, and as predicted).

Fungal predictors

ID	%IncMSE	IncNodePurity	Kingdom	Taxonomy	Isolate	Isolate_percent_id	Isolate_query_cover	glbrc	model_fit_class
OTU_227	0.0580	5.0413	Fungi	227-Helotiales	Leotiomycetes_sp.|GLBRC290	97.238	91	yes	As predicted
OTU_282	0.0552	1.9023	Fungi	282-Tetracladium furcatum	NA	0	0	no	As predicted
OTU_772	0.0465	2.7396	Fungi	772-Glomus sp.	NA	0	0	no	As predicted
OTU_12	0.0424	4.5877	Fungi	12-Cercophora sp.	NA	0	0	no	Below prediction
OTU_109	0.0386	5.2705	Fungi	109-Striaticonidium brachysporum	Myrothecium_cinctum|GLBRC259	99.408	84	yes	Below prediction
OTU_172	0.0382	2.1751	Fungi	172-Ascomycota	NA	0	0	no	Above prediction
OTU_1534	0.0381	3.5519	Fungi	1534-Glomus aggregatum	NA	0	0	no	Above prediction
OTU_57	0.0348	1.6674	Fungi	57-Fusarium sp.	Fusarium_solani	99.5	100	yes	Below prediction
OTU_323	0.0325	4.9919	Fungi	323-Rhizophagus irregularis	NA	0	0	no	Below prediction
OTU_5	0.0322	4.3066	Fungi	5-Pleosporales	Dothideomycetes_sp.|GLBRC458	100	94	yes	Below prediction
OTU_60	0.0320	4.3260	Fungi	60-Helotiales	NA	0	0	no	As predicted
OTU_22	0.0319	2.5416	Fungi	22-Pleosporales	Uncultured_ascomycete|GLBRC490	100	80	yes	Below prediction
OTU_5570	0.0304	1.6504	Fungi	5570-Rhizophagus irregularis	NA	0	0	no	Above prediction
OTU_191	0.0301	4.1826	Fungi	191-Glomus sp.	NA	0	0	no	As predicted
OTU_67	0.0294	2.4478	Fungi	67-Sordariales	NA	0	0	no	Below prediction
OTU_3189	0.0278	2.8490	Fungi	258-Glomus cf. macrocarpum	NA	0	0	no	Above prediction
OTU_10512	0.0246	3.2226	Fungi	10512-Septoglomus sp.	NA	0	0	no	Above prediction
OTU_10	0.0246	3.4734	Fungi	10-Zopfiella sp.	Sordariomycetes_sp.|GLBRC278	100	100	yes	Below prediction
OTU_492	0.0242	3.4713	Fungi	492-Rhizophagus irregularis	NA	0	0	no	Above prediction
OTU_26	0.0230	3.5245	Fungi	26-Periconia sp.	Uncultured_Periconia|GLBRC592	100	93	yes	Below prediction
OTU_258	0.0229	3.2400	Fungi	258-Glomus sp.	NA	0	0	no	Above prediction
OTU_108	0.0227	2.1310	Fungi	108-Ascomycota	NA	0	0	no	Above prediction
OTU_139	0.0224	1.5191	Fungi	139-Sordariomycetes	Uncultured_fungus|GLBRC268	99.435	89	yes	Below prediction
OTU_677	0.0217	2.9169	Fungi	677-Glomeraceae	NA	0	0	no	Above prediction
OTU_1559	0.0213	3.6375	Fungi	1559-Glomeraceae	NA	0	0	no	Above prediction
OTU_126	0.0210	2.4695	Fungi	191-Knufia sp.	NA	0	0	no	Above prediction
OTU_10071	0.0209	3.1779	Fungi	10071-Rhizophagus sp.	NA	0	0	no	Above prediction
OTU_725	0.0198	1.2634	Fungi	725-Glomus sp.	NA	0	0	no	As predicted
OTU_3969	0.0176	1.5909	Fungi	3969-Glomeraceae	NA	0	0	no	Above prediction
OTU_1183	0.0170	1.2640	Fungi	1183-Glomus sp.	NA	0	0	no	Above prediction
OTU_1	0.0165	2.9132	Fungi	1-Setophoma sp.	Setophoma_terrestris|GLBRC188	100	94	yes	As predicted
OTU_77	0.0163	2.4120	Fungi	77-Sordariomycetes	NA	0	0	no	Above prediction
OTU_10558	0.0162	1.4502	Fungi	10558-Glomus sp.	NA	0	0	no	Above prediction
OTU_2848	0.0160	1.9213	Fungi	2848-Glomeraceae	NA	0	0	no	Above prediction
OTU_318	0.0148	2.0304	Fungi	318-Glomus sp.	NA	0	0	no	Above prediction
OTU_2313	0.0142	2.5175	Fungi	2313-Glomus sp.	NA	0	0	no	Above prediction
OTU_4506	0.0139	2.5096	Fungi	4506-Glomeraceae	NA	0	0	no	Above prediction
OTU_266	0.0137	1.5121	Fungi	266-Septoglomus viscosum	NA	0	0	no	As predicted
OTU_967	0.0136	1.2626	Fungi	967-Piriformospora sp.	NA	0	0	no	Above prediction
OTU_23	0.0136	2.4023	Fungi	23-Sordariales	Uncultured_Lasiosphaeriaceae|GLBRC292	98.883	89	yes	Below prediction
OTU_346	0.0136	1.4953	Fungi	346-Paraphoma radicina	Leptosphaeria_sp.|GLBRC475	99.371	80	yes	Above prediction
OTU_4264	0.0135	2.1283	Fungi	4264-Glomus sp.	NA	0	0	no	Above prediction
OTU_690	0.0130	2.3183	Fungi	690-Glomus sp.	NA	0	0	no	Above prediction
OTU_27	0.0130	1.7667	Fungi	27-Pseudogymnoascus sp.	Pseudogymnoascus_pannorum|GLBRC338	99.435	89	yes	Below prediction
OTU_7	0.0125	1.1966	Fungi	7-Pyronemataceae	NA	0	0	no	Below prediction
OTU_230	0.0120	1.3040	Fungi	230-Glomus sp.	NA	0	0	no	As predicted
OTU_467	0.0113	1.3978	Fungi	467-Monosporascus eutypoides	Cryptovalsa_rabenhorstii|GLBRC288	100	87	yes	As predicted
OTU_184	0.0092	1.3343	Fungi	184-Chytridiomycota	NA	0	0	no	Above prediction
OTU_361	0.0084	2.2444	Fungi	361-Macrophomina phaseolina	Macrophomina_phaseolina|GLBRC322	98.87	89	yes	Above prediction
OTU_34	0.0078	2.2710	Fungi	34-Clonostachys sp.	Clonostachys_rosea	99.476	96	yes	Below prediction
OTU_10516	0.0065	1.5564	Fungi	10516-Lasiosphaeriaceae	Podospora_australis|GLBRC665	99.459	92	yes	Above prediction
OTU_534	0.0065	1.6900	Fungi	534-Chytridiomycota	NA	0	0	no	Above prediction
OTU_857	0.0058	0.9416	Fungi	857-Polyschema sclerotigenum	NA	0	0	no	Above prediction
OTU_13	0.0056	2.2612	Fungi	13-Talaromyces sp.	Talaromyces_pinophilus|GLBRC206	100	94	yes	Above prediction

Bacterial predictors

ID	%IncMSE	IncNodePurity	Kingdom	Taxonomy	Isolate	Isolate_percent_id	Isolate_query_cover	glbrc	model_fit_class
OTU_700	0.0625	9.2157	Bacteria	700-Blastopirellula sp.	NA	0	0	no	Below prediction
OTU_281	0.0568	4.5970	Bacteria	281-Gaiella sp.	NA	0	0	no	Below prediction
OTU_6048	0.0394	7.5514	Bacteria	6048-Sphingomonadaceae	NA	0	0	no	As predicted
OTU_21604	0.0328	6.5407	Bacteria	21604-Altererythrobacter sp.	NA	0	0	no	Above prediction
OTU_64	0.0262	3.8232	Bacteria	64-Solirubrobacter sp.	NA	0	0	no	As predicted
OTU_427	0.0229	2.2236	Bacteria	427-Actinobacteria	NA	0	0	no	Below prediction
OTU_451	0.0226	3.7379	Bacteria	451-Xanthomonadales	NA	0	0	no	Below prediction
OTU_727	0.0224	3.5407	Bacteria	727-Cand. Moen. glomeromycotorum	NA	0	0	no	Below prediction
OTU_306	0.0219	3.1849	Bacteria	306-Rhizobiales	NA	0	0	no	Below prediction
OTU_2695	0.0208	2.7683	Bacteria	2695-Iamia sp.	NA	0	0	no	Above prediction
OTU_3551	0.0205	3.1992	Bacteria	3551-Streptomyces sp.	NA	0	0	no	As predicted
OTU_210	0.0203	3.0846	Bacteria	210-Pseudoflavitalea sp.	NA	0	0	no	Below prediction
OTU_7284	0.0192	2.2478	Bacteria	7284-Gemmatimonadetes	NA	0	0	no	Below prediction
OTU_694	0.0186	2.1232	Bacteria	694-Prosthecomicrobium sp.	NA	0	0	no	As predicted
OTU_253	0.0176	3.3495	Bacteria	253-Mycobacterium sp.	NA	0	0	no	Below prediction
OTU_638	0.0173	2.3435	Bacteria	638-Firmicutes	NA	0	0	no	Below prediction
OTU_200	0.0169	3.3666	Bacteria	200-Pedomicrobium sp.	NA	0	0	no	Below prediction
OTU_232	0.0168	1.9856	Bacteria	232-Cand. Moen. glomeromycotorum	NA	0	0	no	Below prediction
OTU_524	0.0164	4.3888	Bacteria	524-Nocardioides sp.	NA	0	0	no	Above prediction
OTU_709	0.0158	2.1879	Bacteria	709-Bauldia sp.	NA	0	0	no	Below prediction
OTU_2472	0.0136	2.8012	Bacteria	2472-Rhizobium sp.	Rhizobium_sp.|GLBRC895	99.543	88	yes	Below prediction
OTU_568	0.0130	2.0177	Bacteria	568-Acidobacteria	NA	0	0	no	Below prediction
OTU_26	0.0130	4.3969	Bacteria	26-Dysgonomonas sp.	NA	0	0	no	Above prediction
OTU_398	0.0122	3.1086	Bacteria	398-Polaromonas sp.	NA	0	0	no	Below prediction
OTU_4189	0.0122	1.5978	Bacteria	4189-Rhizobiales	NA	0	0	no	Above prediction
OTU_516	0.0120	1.6260	Bacteria	516-Bacteria	NA	0	0	no	Below prediction
OTU_325	0.0116	3.1416	Bacteria	325-Hyphomicrobium sp.	NA	0	0	no	As predicted
OTU_5076	0.0111	3.3740	Bacteria	5076-Acidibacter sp.	NA	0	0	no	Above prediction
OTU_2263	0.0110	1.7489	Bacteria	2263-Acidobacteria	NA	0	0	no	Above prediction
OTU_467	0.0106	3.0859	Bacteria	467-Sphingomonas sp.	NA	0	0	no	Above prediction
OTU_3731	0.0103	2.6939	Bacteria	3731-Bacillus sp.	Bacillus_sp.|GLBRC776	98	100	yes	Below prediction
OTU_796	0.0099	1.8528	Bacteria	796-Methylocystaceae	NA	0	0	no	Below prediction
OTU_8370	0.0097	2.2031	Bacteria	8370-Pseudoxanthomonas sp.	Pseudoxanthomonas_indica|GLBRC997	97.585	83	yes	Below prediction
OTU_5	0.0089	3.4061	Bacteria	5-Streptomyces sp.	NA	0	0	no	As predicted
OTU_754	0.0087	1.0498	Bacteria	754-Gemmatimonas sp.	NA	0	0	no	Below prediction
OTU_26913	0.0082	1.6116	Bacteria	26913-Tagaea sp.	NA	0	0	no	Above prediction
OTU_224	0.0082	1.8382	Bacteria	224-Fimbriimonadaceae	NA	0	0	no	Below prediction
OTU_4834	0.0077	2.7303	Bacteria	4834-Myxococcales	NA	0	0	no	Above prediction
OTU_302	0.0075	1.4708	Bacteria	302-Cellvibrio sp.	NA	0	0	no	Below prediction
OTU_6802	0.0073	1.4712	Bacteria	6802-Bacteria	NA	0	0	no	Above prediction
OTU_25487	0.0071	1.8760	Bacteria	25487-Lautropia sp.	NA	0	0	no	Above prediction
OTU_760	0.0069	1.3007	Bacteria	760-Burkholderiales	NA	0	0	no	Below prediction
OTU_2070	0.0067	2.8386	Bacteria	2070-Sandaracinus sp.	NA	0	0	no	Above prediction
OTU_7726	0.0060	1.7398	Bacteria	7726-Lysobacter sp.	NA	0	0	no	Below prediction
OTU_962	0.0052	0.6992	Bacteria	962-Bacteria	NA	0	0	no	Below prediction
OTU_2597	0.0046	0.8818	Bacteria	2597-Rhizobiales	NA	0	0	no	Above prediction
OTU_450	0.0043	1.0857	Bacteria	450-Chitinophagaceae	NA	0	0	no	Above prediction
OTU_8499	0.0042	1.8529	Bacteria	8499-Gammaproteobacteria	NA	0	0	no	Above prediction
OTU_699	0.0041	0.7651	Bacteria	699-Gemmatimonas sp.	NA	0	0	no	Below prediction
OTU_6199	0.0039	0.4879	Bacteria	6199-Bacteria	NA	0	0	no	Above prediction
OTU_20144	0.0036	1.0840	Bacteria	20144-Bacteria	NA	0	0	no	Below prediction
OTU_5225	0.0031	1.2906	Bacteria	5225-Candidatus magasanikbacteria	NA	0	0	no	Above prediction

B) Multiple linear regression. We run linear regression models to predict switchgrass arial plant biomass using the most important OTU selected by the Random Forest models, as predictors.

Fungal predictors

```
Coefficients:
      Estimate    Std. Error  t value     Pr(>|t|)
(Intercept) 1.9950846   0.0833018   23.95 <2.00E-16   ***
OTU_3551    -0.0045518  0.0012911   -3.526      0.000539    ***
OTU_6048    -0.0022031  0.0010145   -2.172      0.031223    *
OTU_210     0.0005875   0.0003395   1.73  0.085326    .
OTU_754     -0.065642   0.0360855   -1.819      0.07061     .
OTU_7284    0.0119521   0.0058194   2.054 0.041478    *
OTU_638     0.0040913   0.0014617   2.799 0.005702    **
OTU_451     -0.0242367  0.008064    -3.006      0.003041    **
OTU_796     -0.0036794  0.0019007   -1.936      0.054503    .
OTU_727     0.0007919   0.0003843   2.061 0.040799    *
OTU_962     0.009153    0.0055737   1.642 0.102345
OTU_700     0.153514    0.0285181   5.383 2.33E-07    ***
OTU_699     0.0426953   0.0242912   1.758 0.080556    .
OTU_8499    0.2600693   0.0801629   3.244 0.001411    **
---
Signif. codes:  0 '***' 0.001 '**' 0.01 '*' 0.05 '.' 0.1 ' ' 1
Residual standard error: 0.6357 on 175 degrees of freedom
Multiple R-squared:  0.5065, Adjusted R-squared:  0.467
F-statistic: 12.83 on 14 and 175 DF,  p-value: < 2.2e-16
```

Bacterial predictors

```
Coefficients:
      Estimate    Std. Error  t value     Pr(>|t|)
(Intercept) 2.30E+00    1.01E-01    22.845      <2.00E-16   ***
FOTU_5      -2.19E-05   1.21E-05    -1.808      0.072381    .
FOTU_10     -3.33E-05   9.23E-06    -3.607      0.000409    ***
FOTU_13     -1.24E-03   3.78E-04    -3.274      0.001288    **
FOTU_23     -2.72E-05   1.20E-05    -2.271      0.0244      *
FOTU_60     2.64E-04    1.76E-04    1.5   0.135414
FOTU_139    3.47E-04    2.17E-04    1.601 0.111326
FOTU_12     -1.35E-05   7.36E-06    -1.839      0.067643    .
FOTU_2313   9.06E-04    4.64E-04    1.954 0.052345    .
FOTU_109    1.02E-04    4.24E-05    2.414 0.016847    *
FOTU_230    5.13E-04    2.55E-04    2.015 0.045501    *
FOTU_690    -4.34E-03   1.22E-03    -3.558      0.000486    ***
FOTU_1559   1.21E-03    4.73E-04    2.56  0.011351    *
FOTU_318    -2.87E-03   1.74E-03    -1.645      0.101787
FOTU_772    -7.34E-03   2.67E-03    -2.747      0.006666    **
FOTU_10512  -2.04E-02   5.91E-03    -3.452      0.000706    ***
FOTU_10558  6.00E-02    2.07E-02    2.906 0.004155    **
FOTU_266    1.55E-03    6.71E-04    2.301 0.022605    *
FOTU_282    -7.22E-04   2.65E-04    -2.72 0.007218    **
FOTU_184    8.02E-03    5.18E-03    1.546 0.123896
FOTU_534    1.18E-03    8.05E-04    1.466 0.144595
FOTU_467    5.47E-04    2.38E-04    2.296 0.022913    *
---
Signif. codes:  0 '***' 0.001 '**' 0.01 '*' 0.05 '.' 0.1 ' ' 1
Residual standard error: 0.6235 on 167 degrees of freedom
Multiple R-squared:  0.5469, Adjusted R-squared:  0.4872
F-statistic: 9.163 on 22 and 167 DF,  p-value: < 2.2e-16
```
