# Supplementary material for: Soil Origin and Plant Genotype Modulate Switchgrass Aboveground Productivity and Root Microbiome Assembly
Source: mBio. 2022 Apr 6;13(2):e00079-22. doi: 10.1128/mbio.00079-22 (PMC9040762; doi:10.1128/mbio.00079-22)
Supplement: FIG S4 [file mbio.00079-22-sf004.pdf]

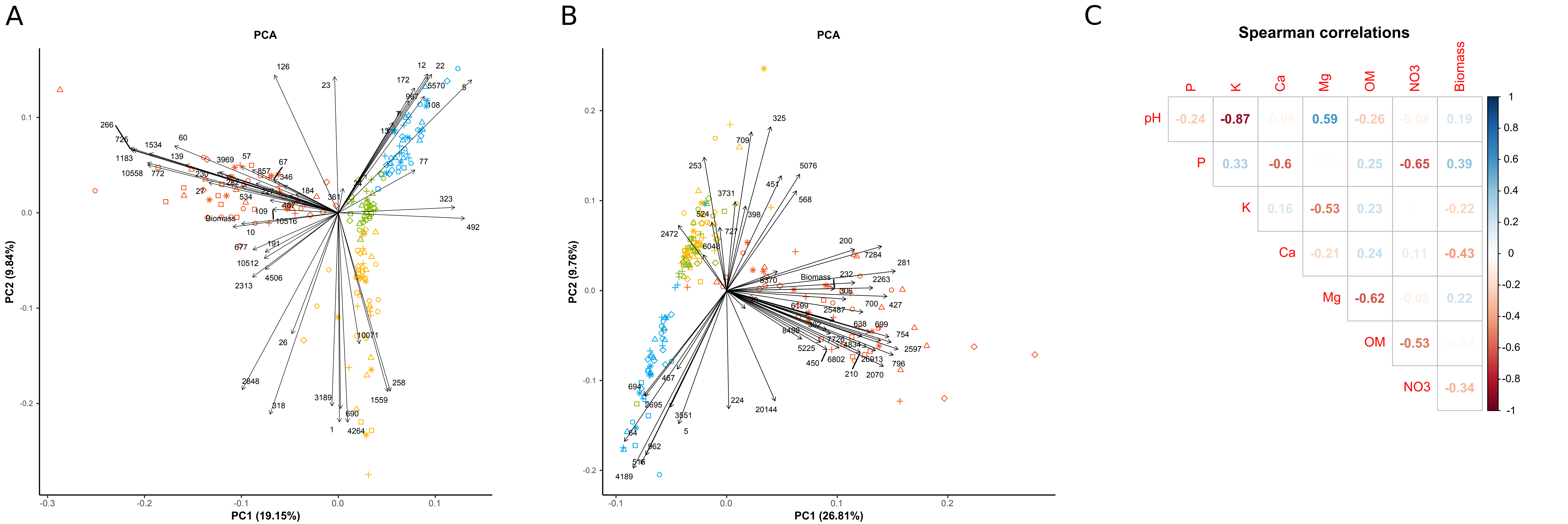

A
PCA
PC1 (19.15%)
PC2 (9.84%)
Biomass
B
PCA
PC1 (26.81%)
PC2 (9.76%)
Biomass
C
Spearman correlations
P
K
Ca
Mg
OM
NO3
Biomass
pH
-0.24
-0.87
-0.06
0.59
-0.26
-0.08
0.19
0.33
-0.6
0.25
-0.65
0.39
0.16
-0.53
0.23
-0.22
-0.21
0.24
0.11
-0.43
-0.62
-0.08
0.22
-0.53
-0.34
1
0.8
0.6
0.4
0.2
0
-0.2
-0.4
-0.6
-0.8
-1
