# Supplementary material for: Soil Origin and Plant Genotype Modulate Switchgrass Aboveground Productivity and Root Microbiome Assembly
Source: mBio. 2022 Apr 6;13(2):e00079-22. doi: 10.1128/mbio.00079-22 (PMC9040762; doi:10.1128/mbio.00079-22)
Supplement: TABLE S4 [file mbio.00079-22-st004.pdf]

Table S4. Mantel test correlation between fungal communities and plant phenotype traits.

```
Mantel statistic based on Spearman's rank correlation rho

Call:
mantel(xdis = otus.bray.s.f, ydis = m.ITS.pheno, method = "spear",
permutations = 999, strata = NULL, na.rm = FALSE, parallel =
getOption("mc.cores"))

Mantel statistic r: 0.1438
      Significance: 0.001

Upper quantiles of permutations (null model):
   90%    95%  97.5%    99%
0.0321 0.0412 0.0490 0.0588
Permutation: free
Number of permutations: 999
```

Fungal communities vs. soil chemical properties:

```
Mantel statistic based on Spearman's rank correlation rho

Call:
mantel(xdis = otus.bray.s.f, ydis = m.ITS.chem, method = "spear",
permutations = 999, strata = NULL, na.rm = FALSE, parallel =
getOption("mc.cores"))

Mantel statistic r: 0.2904
      Significance: 0.001

Upper quantiles of permutations (null model):
   90%    95%  97.5%    99%
0.0196 0.0260 0.0320 0.0374
Permutation: free
Number of permutations: 999
```

Bacterial communities vs. plant phenotype traits:

```
Mantel statistic based on Spearman's rank correlation rho

Call:
mantel(xdis = otus.bray.s, ydis = m.16s.pheno, method = "spear",
permutations = 999, strata = NULL, na.rm = FALSE, parallel =
getOption("mc.cores"))

Mantel statistic r: 0.171
      Significance: 0.001

Upper quantiles of permutations (null model):
```

```
   90%    95%  97.5%    99% 
0.0324 0.0397 0.0449 0.0512 
Permutation: free
Number of permutations: 999
```

Fungal communities vs. soil chemical properties:

```
Mantel statistic based on Spearman's rank correlation rho 

Call:
mantel(xdis = otus.bray.s, ydis = m.16s.chem, method = "spear", 
permutations = 999, strata = NULL, na.rm = FALSE, parallel = 
getOption("mc.cores")) 

Mantel statistic r: 0.3078 
      Significance: 0.001 

Upper quantiles of permutations (null model):
   90%    95%  97.5%    99% 
0.0200 0.0256 0.0312 0.0362 
Permutation: free
Number of permutations: 999
```

Plant phenotype traits vs. soil chemical properties:

```
Mantel statistic based on Spearman's rank correlation rho 

Call:
mantel(xdis = m.16s.pheno, ydis = m.16s.chem, method = "spear", 
permutations = 999, strata = NULL, na.rm = FALSE, parallel = 
getOption("mc.cores")) 

Mantel statistic r: 0.07552 
      Significance: 0.001 

Upper quantiles of permutations (null model):
   90%    95%  97.5%    99% 
0.0223 0.0304 0.0380 0.0459 
Permutation: free
Number of permutations: 999
```
