# Supplementary material for: Soil Origin and Plant Genotype Modulate Switchgrass Aboveground Productivity and Root Microbiome Assembly
Source: mBio. 2022 Apr 6;13(2):e00079-22. doi: 10.1128/mbio.00079-22 (PMC9040762; doi:10.1128/mbio.00079-22)
Supplement: TABLE S5 [file mbio.00079-22-st005.pdf]

Site	Soil Taxonomy	Soil type	Location	coordinates
Lux Arbor	Typic Hapludalf (Alfisol)	Well-drained	Hickory Corners, MI	42.4764 N -85.4519 W
Lake City	Oxyaquic Haplorthod (Spodosol)	moderately well-drained	Lake City, MI	44.2961 N -85.1996 W
Rhineland	Entic Haplorthod (Spodosol)	well-drained	Rhinelander, WI	45.6656 N -89.2180 W
Hancock	Typic Udipsamment (Entisol)	excessively drained	Hancock, WI	44.1194 N -89.5338 W

Table S5. General characteristics, taxonomy, and location of the soils used to build the microcosms.
